# Matching excellence: Oxford Nanopore Technologies’ rise to parity with Pacific Biosciences in genome reconstruction of non-model bacterium with high G+C content

**DOI:** 10.1099/mgen.0.001316

**Published:** 2024-11-11

**Authors:** Axel Soto-Serrano, Wenwen Li, Farhad M. Panah, Yan Hui, Pablo Atienza, Alexey Fomenkov, Richard J. Roberts, Paulina Deptula, Lukasz Krych

**Affiliations:** 1 Section for Food Microbiology, Gut Health and Fermentation, Department of Food Science, University of Copenhagen, Rolighedsvej 26, 1958 Frederiksberg C, Denmark; 2 New England Biolabs, Ipswich, MA 01938-2723, USA

**Keywords:** genome reconstruction, long-read sequencing, single-molecule sequencing

## Abstract

The reconstruction of complete bacterial genomes is essential for microbial research, offering insights into genetic content, ontology and regulation. While Pacific Biosciences (PacBio) provides high-quality genomes, its cost remains a limitation. Oxford Nanopore Technologies (ONT) offers long reads at a lower cost, yet its error rate raises scepticism. Recent ONT advancements, such as new Flow cells (R10.4.1), chemistry (V14) and duplex mode, improve data quality. Our study compares ONT with PacBio and Illumina, including hybrid data. We used *Propionibacterium freudenreichii*, a bacterium with a genome known for being difficult to reconstruct. By combining data from ONT’s Native Barcoding and a custom-developed BARSEQ method, we achieved high-quality, near-perfect genome assemblies. Our findings demonstrate, for the first time, that the combination of nanopore-only long-native with shorter PCR DNA reads (~3 kb) results in high-quality genome reconstruction, comparable to hybrid data assembly from two sequencing platforms. This endorses ONT as a cost-effective, stand-alone strategy for bacterial genome reconstruction. Additionally, we compared methylated motif detection between PacBio and ONT R10.4.1 data, showing that results comparable to PacBio are achievable using ONT, especially when utilizing the advanced Nanomotif tool.

## Data availability

Raw sequences are available at the National Center for Biotechnology Information (NCBI) under the BioProject accession numbers PRJNA1070974 and PRJNA772095. Sequence Read Archive accession numbers are included in Table S4 (available in the online version of this article). All analyses from raw reads to post-assembly quality controls and generated plots were done based on a custom workflow deposited on GitHub (https://github.com/farhadm1990/bactflow). The Pacific Biosciences-generated genome sequences are available at NCBI with accession numbers CP085639 for TL29, CP085640 for TL19 and CP085641 for TL110.

**Impact Statement**

The introduction of next-generation sequencing technologies in 2005 revolutionized the field of microbial genomics. Since then, various sequencing technologies and strategies, including advanced DNA library preparations and assembly methods, have been developed. The main sequencing technologies – Illumina, Oxford Nanopore Technologies (ONT) and Pacific Biosciences (PacBio) – each offer unique advantages and limitations. Researchers have been facing a choice between high-quality, expensive complete genome sequencing (PacBio), cost-effective sequencing with near-perfect quality but incomplete genomes (Illumina) or cost-effective sequencing with nearly complete genomes but higher error rates (ONT).

A widely adopted solution to this dilemma is hybrid assembly, which combines ONT long reads with accurate Illumina short reads. This approach maintains affordable sequencing costs; however, it increases analysis complexity and requires multiple sequencing platforms and library preparation protocols. Nevertheless, recent improvements in ONT data quality have reduced the benefits of Illumina correction.

Our study evaluates the performance of the latest ONT Flow cells, chemistry and library preparation strategies. For the first time, we present data combining nanopore-only long-native and shorter-PCR reads in a peer-reviewed paper, demonstrating optimal results for difficult-to-reconstruct bacterial strains. Additionally, we performed methylation motif calling using ONT and PacBio data. DNA methylation is critically important for gene expression regulation (epigenetics), phage resistance and genome editing, among other functions. While tools for methylation detection using the latest ONT Flow cells remain immature, our work provides data obtained with state-of-the-art analysis tools, showing their current capabilities and limitations.

This research highlights the advancements in ONT technology, emphasizing its potential to deliver high-quality, cost-effective genomic data, thereby pushing the boundaries of microbial genomics and its applications.

## Introduction

The reconstruction of complete, high-quality bacterial genomes is crucial for understanding bacterial diversity, evolution and ecological roles. Studying full bacterial genomes provides critical insights into the basis of pathogenicity, antibiotic resistance and other virulence factors [1,2]. For the past two decades, next-generation sequencing platforms have dominated and revolutionized microbial genomics research, allowing accurate and cost-effective sequencing. However, their main limitation, short-read sequences, often hinders the recovery and assembly of complete, circular genomes. For example, the widely used Illumina technology delivers high-accuracy reads (>99 %) but only short fragments of 500 bp or shorter [3], compromising adequate full genome assembly when repeat regions are larger than those fragments [4,5].

The advent of third-generation sequencing platforms capable of producing long reads, such as Pacific Biosciences (PacBio) and Oxford Nanopore Technologies (ONT), enabled reconstruction of complete genomes, though initially both technologies suffered from significantly high error rates (~10–15 %) [6,7] on a single-molecule level, severely affecting the quality of recovered genomes. PacBio was the first third-generation sequencing that significantly improved the quality, reaching today 99.999% consensus accuracy according to the manufacturer. Although the recovery of high-quality, complete genomes became possible on a single platform, data generation with this technology remains very costly (Table S1).

ONT struggled longer with the error on a single-molecule level, particularly in homopolymer regions, i.e. short sequences of repeated nucleotides, where deletion errors often lead to underestimation of homopolymer length, especially when these exceed four to five bases. Additional sources of error include short tandem repeats, high G+C content, substitution errors in A and T and DNA methylation [8]. In this context, employing hybrid assembly, which combines data from different sequencing technologies, has been a widely used solution to achieve complete and accurate sequences. One of the most extensively utilized tools for this purpose is Unicycler [3], which constructs assembly graphs from the cost-effective, accurate Illumina reads and subsequently bridges assembly gaps using lower-quality long-read data from a third-generation sequencing platform. This approach is time-consuming and costly due to the high initial investment and need for trained personnel for library preparation and data generation on two different sequencing platforms.

Despite enduring challenges, ONT has made great efforts to reduce error rates. In 2023, ONT introduced important upgrades in chemistry (V14), Flow cells (R10.4.1) and duplex mode. Furthermore, new basecalling algorithms (e.g. Dorado v0.7) are constantly being released, and today the company reports accuracy above 99% [9]. This increase in accuracy has also supported the development of hybrid approaches, where long-read-first assemblies are generated and subsequently polished with short reads using tools such as Pypolca [10,11] or Polypolish [11,12]. These methods yield better results in terms of accuracy and contiguity compared to short-read-first approaches like Unicycler [13,14].

ONT offers the most cost-effective sequencing platform on the market, with a broad portfolio of Flow cells and library preparation strategies. Furthermore, it allows DNA sequencing and library preparation without PCR amplification, enabling the study of base methylations, and generates the longest reads by far, with the potential to reach megabases [15]. Moreover, news about the light sensitivity of V14 chemistry (August 2023) led to an instant adjustment in all protocols, requiring ONT libraries to be prepared, loaded and sequenced in the dark. This was speculated to improve the throughput of long-read sequencing up to 141% and amplicon sequencing even up to 469% [16], depending on the device, though official peer-reviewed data on that and their potential impact on quality are not yet available.

Despite many advantages of ONT, it remains unclear to what extent different library preparation strategies, the new chemistry and the newest Flow cell have in fact reduced the error rate. In this paper, we compared sequencing data previously generated on the Illumina NextSeq platform, ONT MinION (R9.4.1) and PacBio RSII, to data generated by the newest ONT Flow cells R10.4.1, utilizing different library preparation methods (Native Barcoding, Rapid Barcoding and the custom-developed BARSEQ), as well as assembly strategies for three strains of Actinomycetota (formerly Actinobacterium), *Propionibacterium freudenreichii*. The genomes of these bacteria have proven difficult to reconstruct in the past due to their high G+C content, regions of repeated sequences and massive genome rearrangements [17,18]. To assess the resulting assemblies, we used CheckM-based genome completeness [19], whole-genome variant calling, gene annotation comparisons and methylation motif recovery.

Finally, we demonstrate that high-quality genomes can be recovered from ONT data alone, especially by complementing Native DNA (with methylations) with a PCR-enriched library (without methylations). To accommodate this, we developed BARSEQ (barcode-amplified random sequencing), a cost-effective, transposase-free DNA enrichment method. BARSEQ incorporates sample-specific barcodes during PCR, reducing costs and allowing pooled libraries to be sequenced alongside Native barcoded samples on a single ONT platform to reconstruct near-perfect bacterial genomes.

## Methods

### Bacteria growth and genomic DNA extraction

**Fig. 1.**
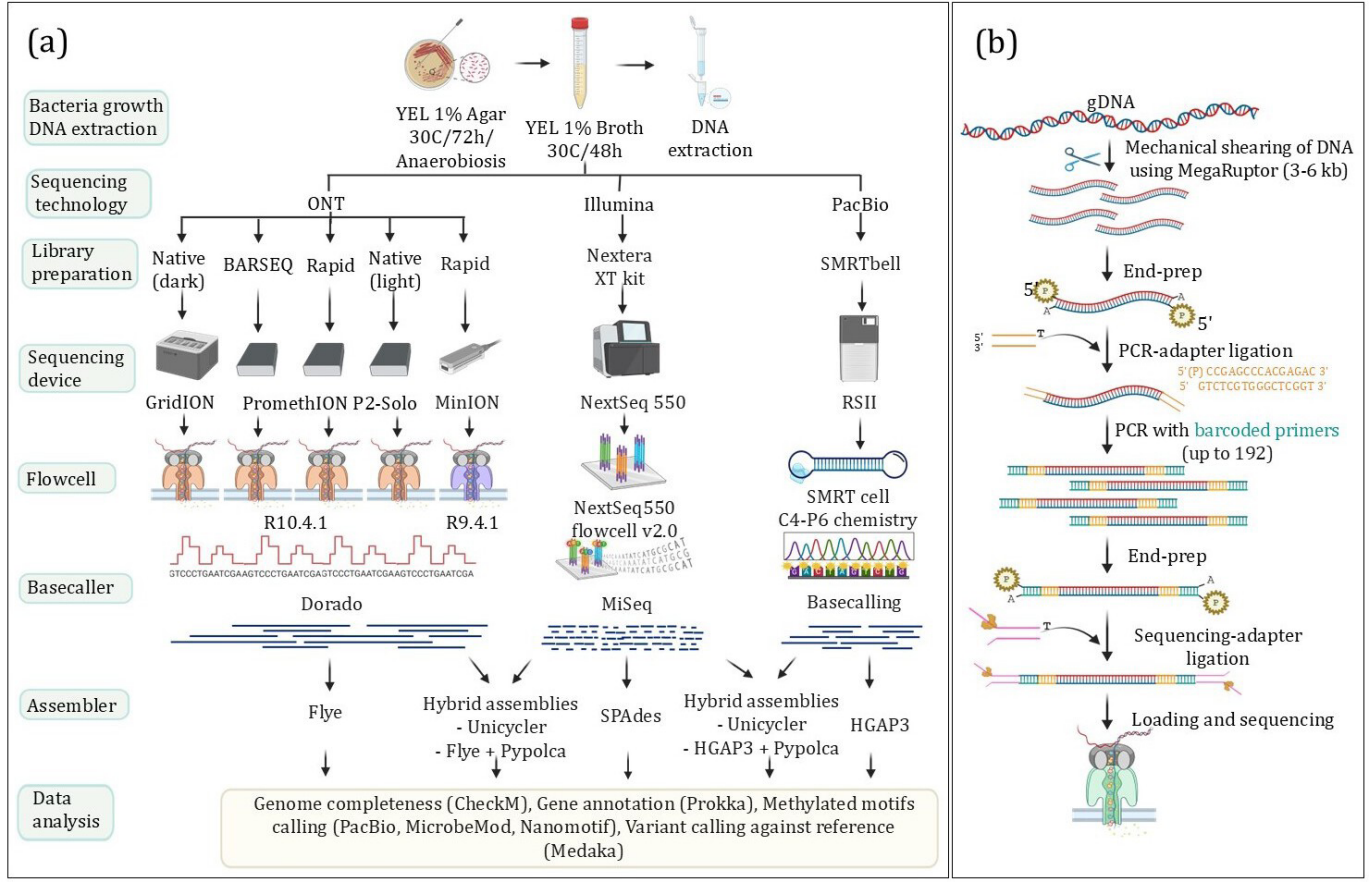
(**a**) Workflow of the experiments. Genomes of three *P. freudenreichii* strains were reconstructed using Illumina NextSeq, PacBio RSII and ONT R9.4.1 and R10.4.1 platforms. Various library preparation strategies (Native Barcoding, Rapid Barcoding and custom BARSEQ) and assembly methods were tested. (**b**) Overview of the BARSEQ protocol. Genomic DNA is sheared and subjected to ligation with palindromic adapters. Ligated primer-binding sites are used for PCR amplification with primers including custom barcodes. The pooled, barcoded DNA is purified and subjected to 1D genomic DNA library preparation by ligation protocol and sequenced with ONT. Figure created with BioRender.

*P. freudenreichii* TL110, TL19 and TL29 were streaked from glycerol (15%) stocks and grown anaerobically (Anaerogen; Thermo Fisher, Waltham, MA, USA) first on yeast extract lactate (YEL) agar [20] for 4 days and then in YEL broth for 48 h, both at 30 °C. The bacterial cells were subsequently centrifuged at 4402 ***g*** for 10 min using a 5920 R centrifuge (Eppendorf, Hamburg, Germany), washed in 1 ml 0.9% NaCl and subsequently centrifuged again at 13 800 ***g*** for 3 min using a Micro Star 17R microfuge (VWR, Radnor, PA, USA). The resulting cell pellets were used for genomic DNA extraction with a Bead-Beat Micro AX Gravity kit following the manufacturer’s instructions (cat #106–100 M1; A&A Biotechnology, Gdynia, Poland) for ONT sequencing on R10.4.1 Flow cells and with MagAttract HMW DNA kit (Qiagen, Germantown, MD, USA) for Illumina, PacBio and ONT sequencing with R9.4.1 Flow cells. Genomic DNA was quantified using a Qubit 4 fluorometer (Thermo Fisher). The experimental workflow is summarized in Fig. 1a.

### DNA library preparation and sequencing

#### Rapid Barcoding for ONT R10.4.1/V14 chemistry

DNA concentration was normalized to 20 ng µl^−1^ (200 ng total) and subjected to library preparation using the Rapid Barcoding Sequencing protocol, following the manufacturer’s instructions (SQK-RBK114.24, version: RBK_9176_v114_revG_27Nov2022; ONT, Oxford, UK). The sequencing was performed on the PromethION 2 Solo (ONT) sequencing platform connected to a custom-build computing station and basecalled with Dorado v0.7.1, using the dna_r10.4.1_e8.2_400bps_sup@v5.0.0 basecalling model.

For R9.4.1 Rapid sequencing, the DNA concentration was normalized to 53.3 ng µl^−1^ (400 ng total) and subjected to library preparation using the Rapid Barcoding Sequencing protocol (SQK-RBK004, version: RBK_9054_v2_revK_14Aug2019; ONT), following the manufacturer’s instructions. The sequencing was performed on the MinION platform (ONT). Data acquisition was performed with MinKNOW 23.07.15.

#### Native Barcoding for ONT R10.4.1/V14 chemistry

DNA concentration was normalized to 33.33 ng µl^−1^ (400 ng total) and subjected to library preparation using the Native Barcoding Sequencing protocol (SQK-NBD114.24, version: NBE_9169_V144_revN_ 15Sep2022; ONT), following the manufacturer’s instructions. The sequencing was performed on GridION and PromethION 2 Solo platforms (ONT), utilizing R10.4.1 Flow cells. Duplex reads were selected using Duplex-Tools (https://pypi.org/project/duplex-tools/ v0.3.3); both non-duplex and duplex data were further basecalled with Dorado v0.7.1, using the dna_r10.4.1_e8.2_400bps_sup@v5.0.0 basecalling model.

### BARSEQ for ONT R10.4.1/V14 chemistry

DNA concentration was normalized to 50 ng µl^−1^ and the DNA was sheared to ~3 kbp fragments using Megaruptor 2 (Diagenode, Seraing, Belgium) with default fragmentation settings. Shearing quality was estimated with TapeStation 4200 (Agilent Technologies, Santa Clara, CA, USA). The sheared DNA was subjected to ligation with palindromic adapters and PCR amplification using primers with custom barcodes. The sequences of the adapters and barcodes are provided in Table S2. Sheared DNA (25 µl of concentration 0.5 ng µl^−1^) was mixed with 1.75 µl of Ultra II End-prep Reaction Buffer and 1.5 µl of Ultra II End-prep Enzyme Mix (cat #E7546; New England Biolabs, Ipswich, MA, USA) and incubated at 20 °C for 5 min and 65 °C for 5 min. After the End-prep reaction, the DNA was purified with 30 µl of AMPure XP beads according to the manufacturer’s instructions (Beckman Coulter Genomic, CA, USA) and resuspended in 11 µl of nuclease-free water.

The custom double-stranded adapter containing primer-binding site was ligated by mixing 10 µl of NEB TA/Blunt Ligase Master Mix (cat #M0367, New England Biolabs), 2 µl of duplex adapter (1 µM) and 10 µl of End-prepped DNA, and incubated at room temperature for 10 min. The sequence of the duplex adapter is as follows: first strand, 5′(5phos)-CCGAGCCCACGAGAC-3′; second strand, 5′-GTCTCGTGGGCTCGGT-3′. After the ligation, the reaction was purified with AMPure XP beads according to the manufacturer’s manual (Beckman Coulter Genomic, Brea, CA, USA) and resuspended in 12 µl of nuclease-free water.

Finally, the DNA containing the ligated primer-binding site was subjected to the PCR reaction. The PCR mix consisted of 12 µl of PCRBIO Ultra Mix (PCR Biosystems Ltd., London, UK), 2 µl of barcode primers (5 µM; see Table S2 for details) and 11 µl of purified DNA with adapters. The PCR thermal conditions were as follows: denaturation at 95 °C for 3 min; 18 cycles of 95 °C for 30 s, 55 °C for 30 s and 69 °C for 5 min, followed by a final elongation at 72 °C for 4 min. The final PCR products were pooled and purified using AMPure XP beads (Beckman Coulter Genomic) and resuspended in 20 µl of nuclease-free water.

Lastly, purified amplicons were subjected to 1D genomic DNA library preparation by the ligation protocol (SQK-LSK114; ONT) to complete library preparation for sequencing on the PromethION 2 Solo platform (ONT). Approximately 0.2 µg of amplicons were used for the initial end-prep step. Around 40 ng of the prepared amplicon library was loaded onto a R10.4.1 Flow cell. The reads were basecalled with Dorado v0.7.1, using the dna_r10.4.1_e8.2_400bps_sup@v5.0.0 basecalling model. It is important to note that a similar protocol (ligation sequencing V14 – low input by PCR: SQK-LSK114 and EXP-PBC096) is now commercially available from ONT. However, in our protocol, barcoding occurs during PCR, and the final ligation is performed on the pooled library (see Fig. 1b). More importantly, since BARSEQ uses custom polymerase and oligonucleotides, it allows for a cost reduction of approximately 20-fold.

#### Library preparation and sequencing with PacBio

DNA samples were sheared to an average size of ~15 kbp using the G-tube protocol (Covaris, Woburn, MA, USA). DNA libraries were prepared using a SMRTbell express template prep kit 2.0 (100-938-900; PacBio, Menlo Park, CA, USA) and ligated with a hairpin adapter. Incompletely formed SMRTbell templates were removed by digestion with a combination of exonuclease III and exonuclease VII (New England Biolabs). The qualification and quantification of the SMRTbell libraries were made on a Qubit fluorimeter (Invitrogen, Waltham, MA, USA) and a 2100 Bioanalyzer (Agilent Technologies, Santa Clara, CA, USA). Additional separation of SMRTbell libraries on a gel-based BluePippin instrument (Sage Science, Beverly, MA, USA) was required due to inhibition of sequencing resulting from suspected contamination with high-molecular-weight carbohydrate polymers. Finally, SMRT sequencing was performed using a PacBio RSII (PacBio) sequencer with C4-P6 chemistry (seven to ten SMRT cells with 300 min collection time) based on the protocol for 20 kb SMRTbell library inserts.

#### Nextera tagmentation and sequencing with Illumina

DNA concentration was normalized to 1 ng µl^−1^ and subjected to library preparation using the Nextera XT DNA Library Preparation Kit (Illumina, San Diego, CA, USA) according to the manufacturer’s instructions and sequenced with NextSeq 550 using Mid Output Kit v2.5 (300 cycles, #20 024 905; Illumina) using a NextSeq 550 v2.0 Flow cell.

#### Methylation motif verification with restriction enzymes

Genomic DNA of three *P. freudenreichii* strains (TL110, TL19 and TL29) was subjected to the test with restriction enzymes ApaI and BanII (R0114 and R0119, respectively, by New England Biolabs). The reaction with BanII was assembled as follows: 1× rCutSmart Buffer, 1 µl of BanII enzyme and 100 ng of genomic DNA of the respective strain and filled up to 50 µl with nuclease-free water. The incubation was conducted at 37 °C for 1 h followed by 80 °C for 10 min. The reaction with ApaI included 1× rCutSmart Buffer, 1 µl of ApaI enzyme, 500 ng of genomic DNA of the respective strain and filled up to 50 µl with nuclease-free water. The incubation was conducted at 37 °C for 1 h followed by 65 °C for 10 min. DNA after digestion was evaluated using the TapeStation 4200 (Agilent Technologies).

### Data analysis and bioinformatics

#### Raw data quality assessment

Quality scores of Illumina and ONT reads were assessed utilizing FastQC (v0.12.1) and a custom function (https://github.com/farhadm1990/bactflow), respectively. ONT reads were further filtered using NanoFilt (v2.6.0) [21]. The R10.4.1- and R9.4.1-generated reads were filtered by a quality score of 16 and a length of 1000, and a quality score of 7 and a length of 1000, respectively.

#### Genomes assembly

ONT reads were assembled using Flye [22], which has been demonstrated to be the best *de novo* assembler for long reads [23]. In order to avoid any bias due to differences in read depth for different ONT library preparation strategies, all reads were normalized to 80× coverage for a genome size of 2.7 Mbp utilizing Rasusa v0.7,0 [24], except for strain TL19 with Rapid Barcoding utilizing R9.4.1, where a lower throughput led us to use a coverage of 40×. For the assembly of combined Native and BARSEQ reads, the 80× fastq files derived from both library preparation strategies were concatenated in a single file and subsequently normalized again to 80×. Flye v2.9.1 was used with the --nano-hq and --nano-raw options for all R10.4.1- and R9.4.1-derived data, respectively. All the assembled contigs were subsequently polished with Medaka v1.12.1 (https://github.com/nanoporetech/medaka) using the model r1041_e82_400bps_sup_v5.0.0. However, only the unpolished genomes were used for downstream analysis, as polishing did not consistently have a positive effect on accuracy.

Illumina Nextera 550 reads were assembled using SPAdes v3.15.4 [25] with the --isolate flag for higher sensitivity.

Short-read-first hybrid assembly of ONT and Illumina reads was performed with Unicycler v0.5.0 [3] with a conservative mode. Long-read-first hybrid assembly was performed by polishing the previously obtained Flye v2.9.1 assemblies with Pypolca v0.3.1 [10,11], using the --careful flag.

Finally, PacBio reads were *de novo* assembled using the RS_HGAP_Assembly3 (HGAP3) v2.3.0 program [26] with default quality and read length parameters. All genomes were fixed for their replication start point based on the dnaA gene using Circlator v1.5.5 [27], and genomes with successfully fixed start were considered complete and circular.

Overall assembly quality of all genomes across different techniques and strains was assessed by Quast v5.2.0 (https://github.com/ablab/quast/releases/tag/quast_5.2.0).

#### Generation of reference assemblies

Genome references for the TL110, TL19 and TL29 strains were generated by assembling two sets of data and trying to find consensus among them [28].

First, ONT R10.4.1 Native and PCR (BARSEQ) data were filtered using Filtlong v0.2.1 (https://github.com/rrwick/Filtlong) with the parameters --min_length 1,000, --keep_percent 90 and --target_bases 1 500 000 000, and subsequently concatenated in a single fastq file. Then, an assembly was performed as described by Wick *et al*. [29]. Briefly, the filtered ONT reads were assembled using Trycycler v0.5.4 [30], following the ‘extra-thorough assembly’ instructions provided in Trycycler’s wiki (https://github.com/rrwick/Trycycler/wiki), i.e. utilizing Flye v 2.9.2 [22], Canu v2.2 [31], Raven v1.8.1 [32], miniasm v0.3 [33] + Minipolish v0.1.2 [34], NECAT v20200803 [35] and NextDenovo v2.5.0 [36]/NextPolish v1.4.0 [37]. After generating a consensus assembly with Trycycler, this was further polished twice with Illumina reads, utilizing Polypolish v0.6.0 [11,12] and Pypolca v0.3.1 [10,11], respectively. Both polishers were used with the --careful flag due to the large repeat regions in the *Propionibacterium* genomes [17]. Medaka polishing was not performed since it has been found to reduce accuracy in some cases [11,38].

The second assembly was performed from PacBio RSII obtained reads. The reads data were filtered with Filtlong v0.2.1 (https://github.com/rrwick/Filtlong), with the parameters --min_length 1000, --keep_percent 90 and --target_bases 2 500 000 000. The assembly was performed as described for the ONT data, i.e. using Trycycler v0.5.4 [30,39], following the ‘extra-thorough assembly’ instructions, but including an assembly performed with HGAP3 [26]. Polishing with Illumina reads was performed as described for the ONT data. The assemblies were reoriented to start from *dnaA* utilizing dnaapler [40].

Differences between the assemblies were examined with the compare_assemblies.py script provided in https://github.com/rrwick/Perfect-bacterial-genome-tutorial, utilizing the mappy aligner provided by minimap2 v2.24 [41].

#### Gene annotation and genome completeness

All assembled genomes were annotated using Prokka v1.14.6 [42] with default settings. Additionally, the genome completeness within the known gene sets of the *Propionibacterium* genus was assessed using CheckM v1.2.2 [19]. Both analyses were performed utilizing the Bactflow pipeline (https://github.com/farhadm1990/bactflow). Pan-genome analysis was performed with Roary v3.13.0 [43], and a clustering tree created using the presence/absence of accessory genes, i.e. not common for all the assemblies of each of the strains, was visualized with iTOL v5 [44].

#### Variant calling

Variant calling, i.e. insertions and deletions (indels), as well as substitutions, including transitions and transversions, was performed against the generated reference assemblies. Variant calling was performed using medaka_haploid_variant command in Medaka. Only variants with a minimum Q score of 15 were selected for further analysis.

#### Methylation calling

Methylation calling with Nanomotif v0.4.7 [45] was performed on Native R10.4.1 data. Basecalling was performed by Dorado v0.7.1, utilizing the sup mode, i.e. dna_r10.4.1_e8.2_400bps_sup@v5.0.0 with the --modified-bases-models flag using the models dna_r10.4.1_e8.2_400bps_sup@v5.0.0_6mA@v1 and dna_r10.4.1_e8.2_400bps_sup@v5.0.0_4mC_5mC@v1. Methylated motifs were determined following Nanomotif’s documentation (https://nanomotif.readthedocs.io/en/latest/index.html) using nanomotif find_motifs. Methylation calling for ONT data using MicrobeMod [46] was performed using Native R10.4.1 data following the instructions provided in their GitHub (https://github.com/cultivarium/MicrobeMod) in the moment of the realization of the study, i.e. basecalling was performed by Dorado v0.5.1 utilizing the model dna_r10.4.1_e8.2_400bps_sup@v4.3.0 and the --modified-bases-models flag using the models dna_r10.4.1_e8.2_400bps_sup@v4.3.0_6mA@v2 and res_dna_r10.4.1_ e8.2_400bps_sup@v4.3.0_4mC_5mC@v1. The latter was obtained from the Rerio repository (https://github.com/nanoporetech/rerio). MicrobeMod was used to call the methylations with default settings.

For PacBio data, the SMRT Analysis pipeline (http://www.pacbiodevnet.com/SMRT-Analysis/Software/SMRT-Pipe) was used to determine the epigenetic status of sequenced DNA by identifying the 6mA and 4mC modified motifs [47,49] and further curated at REBASE [50].

For the analysis, only motifs detected as methylated by either PacBio or ONT in >80% of their occurrences in the genomes were considered, given that methylation typically occurs in ratios close to 100% in most cases [51,52]. Motifs classified as ‘ambiguous’ by Nanomotif were also discarded.

## Results

### Raw data quality

The details presenting the raw read data quality generated with Illumina and ONT across various Flow cells and library preparation techniques for TL110, TL19 and TL29 strains are given in Figs S1 and S2.

No considerable differences were observed when comparing the quality of the data obtained utilizing light and dark modes (data not shown), yet an increase in data output was observed. Only data obtained from sequencing in the dark mode were utilized for the downstream analysis. Although the increase in throughput is not straightforward to demonstrate with the limited number of samples presented here, we have observed an increase in the total Flow cell throughput ranging from about 30% to over 100% when comparing multiple R.10.4.1 runs before and after the dark mode on various ONT kits (data not shown).

For V14 chemistry, duplex reads constituted 17–20 % of the Native Barcoding reads sequenced with the R10.4.1 Flow cell in dark mode, as calculated by Dorado. We observed a substantial increase in the quality score of the duplex-paired data compared to non-duplex R10.4.1 Native data (Fig. S1). Additionally, the data recovered using split_pairs (https://pypi.org/project/duplex-tools/0.3.3/) did not improve the quality in comparison to R10.4.1 non-duplex Native data (data not shown), and therefore, it was not used for the downstream analysis.

### Generation of reference assemblies

Assemblies were conducted as described in the Methods section. For ONT data, Trycycler produced single contig genomes of 2 566 320 bp, 2 578 970 bp and 2 566 320 bp for strains TL110, TL19 and TL29, respectively. Polishing with Illumina reads using Polypolish resulted in a 1 bp substitution for TL19 and TL29, and no changes for TL110. The subsequent polishing with Pypolca did not alter any of the assemblies.

For PacBio data, Trycycler assemblies resulted in single contig genomes of 2 566 306, 2 578 825 and 2 566 185 for TL110, TL19 and TL29, respectively. Polishing with Illumina reads using Polypolish introduced 10, 138 and 124 bp insertions. Further polishing with Pypolca added 1, 2, and 3 bp insertions, yielding final genome sizes of 2 566  317, 2 578 965 and 2 566 312 bp for TL110, TL19 and TL29, respectively.

Comparison of the assemblies revealed 3, 5 and 8 differences in TL110, TL19 and TL29, respectively, all within G/C homopolymers of 4–10 bp. Notably, these homopolymers were 1 bp shorter in the PacBio-derived assemblies. Manual examination of the differences (Table S3) indicated that the ONT-derived assemblies were more accurate. Consequently, these assemblies were used as the references for downstream analyses.

### Genome completeness and contiguity

To assess the performance of diverse sequencing and assembly approaches, we examined the genomes of three *P. freudenreichii* strains using data from PacBio RSII, Illumina NextSeq 550 and ONT. Common assemblers – HGAP3, SPAdes, and Flye – were employed for assembling PacBio, Illumina and ONT data, respectively. Additionally, hybrid assemblies utilizing both short-read-first and long-read-first approaches were performed utilizing Unicycler and Flye+Pypolca, respectively. The post-assembly quality overview of *de novo* assembled genomes is given in Table 1.

**Table 1.** Overview of assembly performance parameters for three strains of *P. freudenreichii* using data generated with PacBio, ONT and Illumina using various library preparation strategies and assemblers

Sequencing strategy	Assembler	Contigs	Largest contig	Total length	Reference length	G+C (%)	Contig N50	CDS no.	CDS – ref. CDS
**TL110**									
ONT BARSEQ	Flye	21	316 961	2 565 280	2 566 320	67.29	219 797	2233	5
ONT Native (duplex)	Flye	1	2 566 341	2 566 341	2 566 320	67.33	2 566 341	2262	34
ONT Native	Flye	1	2 566 332	2 566 332	2 566 320	67.33	2 566 332	2249	21
ONT Native+BARSEQ	Flye	1	2 566 315	2 566 315	2 566 320	67.33	2 566 315	2226	−2
ONT Rapid	Flye	1	2 566 058	2 566 058	2 566 320	67.32	2 566 058	2520	292
ONT Rapid (R9.4.1)	Flye	1	2 566 736	2 566 736	2 566 320	67.13	2 566 736	3236	1008
ONT Native (duplex)+Illumina Nextera XT	Flye+Pypolca	1	2 566 320	2 566 320	2 566 320	67.33	2 566 320	2227	−1
ONT Native+Illumina Nextera XT	Flye+Pypolca	1	2 566 320	2 566 320	2 566 320	67.33	2 566 320	2226	−2
ONT Native +BARSEQ+Illumina Nextera XT	Flye+Pypolca	1	2 566 320	2 566 320	2 566 320	67.33	2 566 320	2227	−1
ONT BARSEQ+Illumina Nextera XT	Flye+Pypolca	21	316 961	2 565 281	2 566 320	67.29	219 797	2230	2
ONT Rapid+Illumina Nextera XT	Flye+Pypolca	1	2 566 293	2 566 293	2 566 320	67.33	2 566 293	2235	7
ONT Rapid (R9.4.1)+Illumina Nextera XT	Flye+Pypolca	1	2 566 414	2 566 414	2 566 320	67.32	2 566 414	2294	66
PacBio RSII	HGAP3	1	2 566 312	2 566 312	2 566 320	67.33	2 566 312	2230	2
PacBio RSII+Illumina Nextera XT	HGAP3+Pypolca	1	2 566 318	2 566 318	2 566 320	67.33	2 566 318	2228	0
Illumina Nextera XT	SPAdes	127	150 752	2 535 349	2 566 320	67.29	43 970	2232	4
ONT Native (duplex)+Illumina Nextera XT	Unicycler	3	2 534 276	2 566 183	2 566 320	67.33	2 534 276	2233	5
ONT Native+Illumina Nextera XT	Unicycler	4	2 533 438	2 566 353	2 566 320	67.33	2 533 438	2234	6
ONT Native+BARSEQ+Illumina Nextera XT	Unicycler	3	2 534 274	2 566 181	2 566 320	67.33	2 534 274	2230	2
PacBio RSII+Illumina Nextera XT	Unicycler	3	2 536 295	2 568 202	2 566 320	67.33	2 536 295	2262	34
ONT BARSEQ+Illumina Nextera XT	Unicycler	8	1 242 713	2 521 457	2 566 320	67.39	502 860	2198	−30
ONT Rapid+Illumina Nextera XT	Unicycler	3	2 534 242	2 566 149	2 566 320	67.33	2 534 242	2241	13
ONT Rapid (R9.4.1)+Illumina Nextera XT	Unicycler	4	2 533 518	2 565 712	2 566 320	67.32	2 533 518	2264	36
**TL19**									
ONT Native (duplex)	Flye	1	2 578 970	2 578 970	2 578 970	67.33	2 578 970	2248	−1
ONT Native	Flye	1	2 578 970	2 578 970	2 578 970	67.33	2 578 970	2248	−1
ONT Native+BARSEQ	Flye	1	2 578 969	2 578 969	2 578 970	67.33	2 578 969	2249	0
ONT BARSEQ	Flye	26	423 009	2 581 811	2 578 970	67.20	162 216	2270	21
ONT Rapid	Flye	1	2 578 967	2 578 967	2 578 970	67.33	2 578 967	2248	−1
ONT Rapid (R9.4.1)	Flye	1	2 578 664	2 578 664	2 578 970	67.33	2 578 664	2517	268
ONT Native (duplex)+Illumina Nextera XT	Flye+Pypolca	1	2 578 970	2 578 970	2 578 970	67.33	2 578 970	2248	−1
ONT Native+Illumina Nextera XT	Flye+Pypolca	1	2 578 970	2 578 970	2 578 970	67.33	2 578 970	2248	−1
ONT Native+BARSEQ+Illumina Nextera XT	Flye+Pypolca	1	2 578 970	2 578 970	2 578 970	67.33	2 578 970	2248	−1
ONT BARSEQ+Illumina Nextera XT	Flye+Pypolca	26	423 010	2 581 811	2 578 970	67.20	162 217	2264	15
ONT Rapid+Illumina Nextera XT	Flye+Pypolca	1	2 578 970	2 578 970	2 578 970	67.33	2 578 970	2248	−1
ONT Rapid (R9.4.1)+Illumina Nextera XT	Flye+Pypolca	1	2 578 954	2 578 954	2 578 970	67.33	2 578 954	2253	4
PacBio RSII	HGAP3	1	2 593 456	2 593 456	2 578 970	67.32	2 593 456	2299	50
PacBio RSII+Illumina Nextera XT	HGAP3+Pypolca	1	2 593 547	2 593 547	2 578 970	67.32	2 593 547	2269	20
Illumina Nextera XT	SPAdes	158	68 313	2 545 349	2 578 970	67.27	31 134	2249	0
ONT Native (duplex)+Illumina Nextera XT	Unicycler	2	2 581 105	2 582 113	2 578 970	67.32	2 581 105	2251	2
ONT Native+Illumina Nextera XT	Unicycler	2	2 581 103	2 582 111	2 578 970	67.32	2 581 103	2251	2
ONT Native+BARSEQ+Illumina Nextera XT	Unicycler	2	2 581 103	2 582 111	2 578 970	67.32	2 581 103	2251	2
PacBio RSII+Illumina Nextera XT	Unicycler	2	2 581 133	2 582 141	2 578 970	67.32	2 581 133	2264	15
ONT BARSEQ+Illumina Nextera XT	Unicycler	7	1 585 368	2 562 510	2 578 970	67.35	1 585 368	2237	−12
ONT Rapid+Illumina Nextera XT	Unicycler	2	2 581 098	2 582 106	2 578 970	67.32	2 581 098	2251	2
ONT Rapid (R9.4.1)+Illumina Nextera XT	Unicycler	1	2 497 808	2 497 808	2 578 970	67.33	2 497 808	2201	−48
**TL29**									
ONT Native	Flye	1	2 566 326	2 566 326	2 566 320	67.33	2 566 326	2255	23
ONT Native (duplex)	Flye	1	2 566 332	2 566 332	2 566 320	67.33	2 566 332	2260	28
ONT Native+BARSEQ	Flye	1	2 566 319	2 566 319	2 566 320	67.33	2 566 319	2231	−1
ONT BARSEQ	Flye	21	382 373	2 572 798	2 566 320	67.35	220 588	2260	28
ONT Rapid	Flye	1	2 566 324	2 566 324	2 566 320	67.33	2 566 324	2271	39
ONT Rapid (R9.4.1)	Flye	1	2 566 603	2 566 603	2 566 320	67.14	2 566 603	3174	942
ONT Native (duplex)+Illumina Nextera XT	Flye+Pypolca	1	2 566 320	2 566 320	2 566 320	67.33	2 566 320	2231	−1
ONT Native+Illumina Nextera XT	Flye+Pypolca	1	2 566 321	2 566 321	2 566 320	67.33	2 566 321	2232	0
ONT Native+BARSEQ+Illumina Nextera XT	Flye+Pypolca	1	2 566 320	2 566 320	2 566 320	67.33	2 566 320	2231	−1
ONT BARSEQ+Illumina Nextera XT	Flye+Pypolca	21	382 373	2 572 805	2 566 320	67.35	220 588	2257	25
ONT Rapid+Illumina Nextera XT	Flye+Pypolca	1	2 566 322	2 566 322	2 566 320	67.33	2 566 322	2230	−2
ONT Rapid (R9.4.1)+Illumina Nextera XT	Flye+Pypolca	1	2 566 382	2 566 382	2 566 320	67.33	2 566 382	2279	47
PacBio RSII	HGAP3	1	2 566 309	2 566 309	2 566 320	67.30	2 566 309	2233	1
PacBio RSII+Illumina Nextera XT	HGAP3+Pypolca	1	2 566 318	2 566 318	2 566 320	67.30	2 566 318	2233	1
Illumina Nextera XT	SPAdes	143	89 206	2 535 202	2 566 320	67.28	28 523	2249	17
ONT Native (duplex)+Illumina Nextera XT	Unicycler	3	978 011	2 536 351	2 566 320	67.36	842 555	2205	−27
ONT Native+Illumina Nextera XT	Unicycler	4	978 011	2 519 783	2 566 320	67.36	715 785	2183	−49
ONT Native+BARSEQ+Illumina Nextera XT	Unicycler	3	978 009	2 536 320	2 566 320	67.36	842 526	2207	−25
PacBio RSII+Illumina Nextera XT	Unicycler	3	977 999	2 536 277	2 566 320	67.36	842 497	2210	−22
ONT BARSEQ+Illumina Nextera XT	Unicycler	7	757 376	2 494 356	2 566 320	67.37	429 228	2158	−74
ONT Rapid+Illumina Nextera XT	Unicycler	3	978 008	2 536 309	2 566 320	67.36	842 519	2208	−24
ONT Rapid (R9.4.1)+Illumina Nextera XT	Unicycler	1	2 566 161	2 566 161	2 566 320	67.33	2 566 161	2238	6

**Fig. 2.**
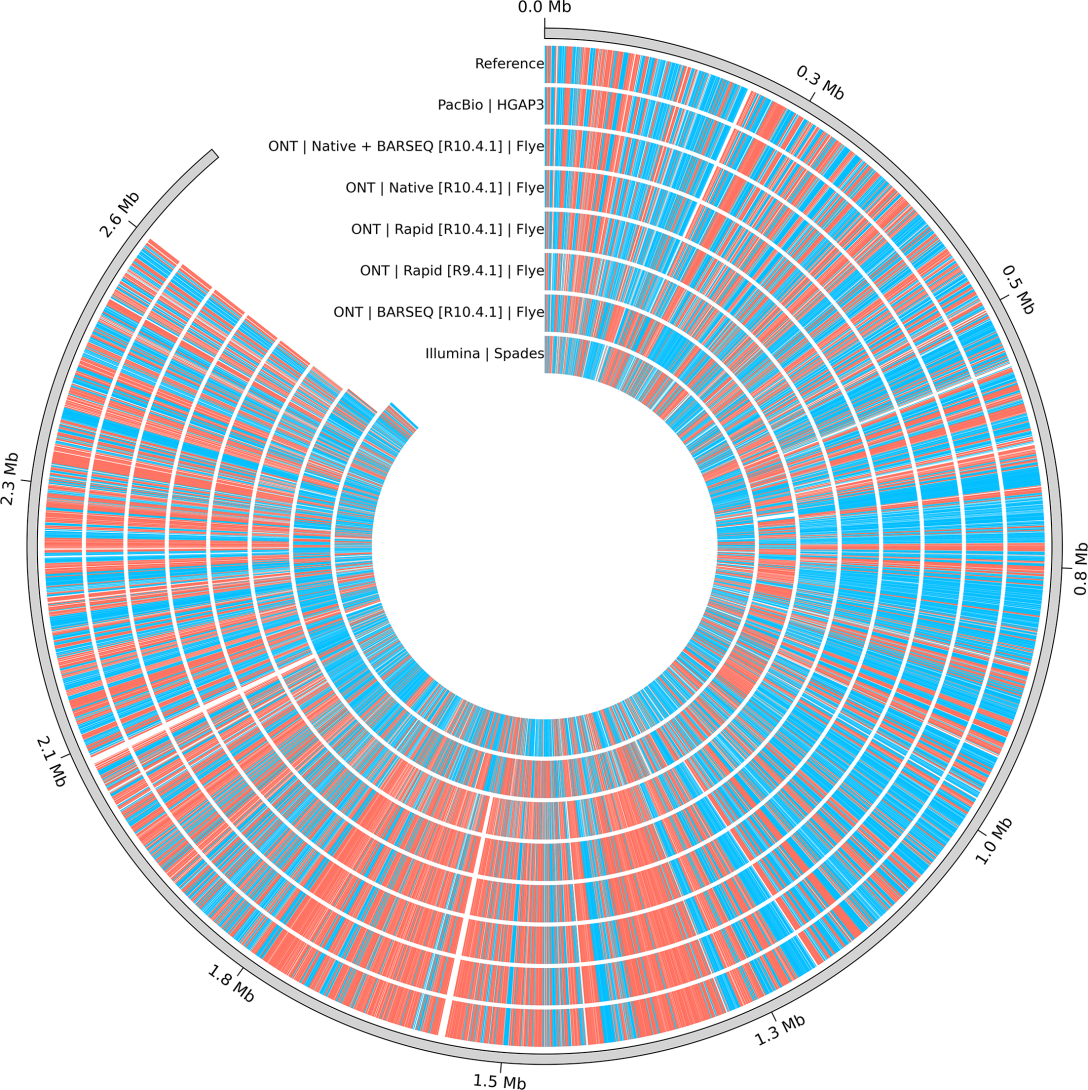
Circularized genome assemblies obtained from a single sequencing technology (ONT, PacBio or Illumina) for the TL29 strain. The reference assembly is placed in the outermost ring. Red and blue stripes correspond to coding sequences (CDS) in the forward and reverse strands, respectively. Circularized genomes for TL110 and TL19 can be found in Fig. S3.

As expected, only data assembled from long-read sequencing strategies where the DNA library was prepared without PCR managed to recover circular single contigs of length >2.5 Mbp, given the absence of plasmids in the studied strains. This is the case for all the PacBio and ONT library preparation methods, except for BARQ, as well as all the long-read-first hybrid assemblies derived from these (Table 1, Figs 2 and S3). The number of contigs in ONT BARSEQ assemblies ranged between 21 and 26. On the other hand, Unicycler short-read-first hybrid assemblies only managed to recover single-contig assemblies when combining Illumina data with ONT R9.4.1 data; however, these were determined to be misassembled in the QUAST analysis, indicating structural errors in the sequence where flanking regions are incorrectly aligned, ordered, or positioned compared to the reference genome. The number of contigs for all the other Unicycler assemblies varied between 2 and 8. The least satisfactory contiguity was observed with Illumina-only data assembled using SPAdes, resulting in 123–158 contigs per strain, with the longest contigs averaging 102 757 bp.

**Fig. 3.**
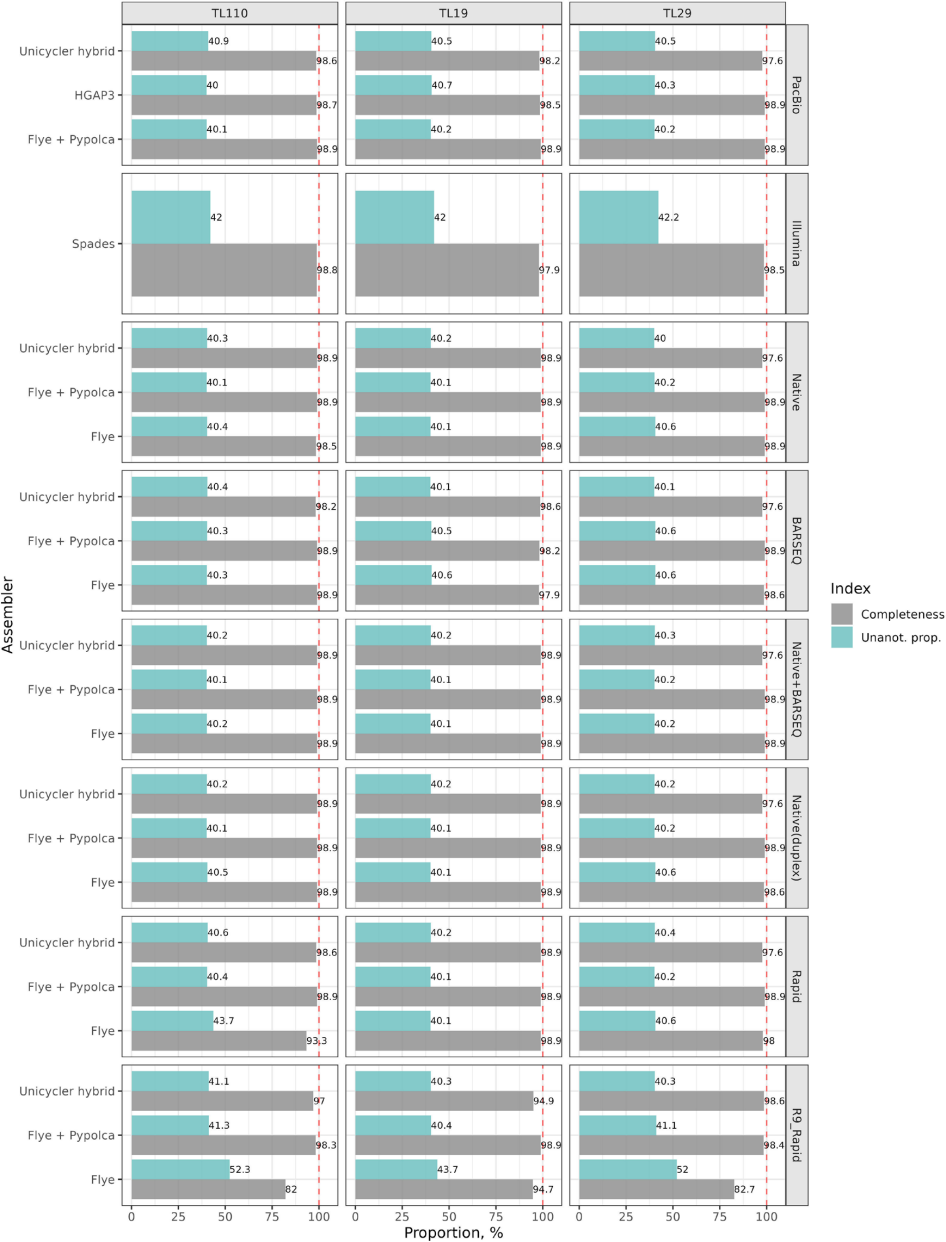
CheckM-based genome completeness and proportion of unannotated genes for the TL110, TL19 and TL29 strains using different sequencing and assembly strategies. Unicycler and Pypolca correspond to short-read-first and long-read-first hybrid assemblies, respectively.

In general, elevated genome completeness CheckM scores (>97 %) could be achieved with all sequencing strategies with Flye, HGAP3 and SPAdes for ONT, PacBio and Illumina, respectively, or through hybrid assemblies utilizing Pypolca and Unicycler (Fig. 3). Exceptions are assemblies generated for TL110 Rapid using Flye and TL19 Rapid (R9.4.1) using both Flye and Unicycler, ranging from 93.31 to 94.92%. Finally, the lowest completeness was observed for TL110 and TL29 Rapid (R9.4.1) assembled with Flye, with completeness ~82%.

### Quality of the assembled genomes: variant calling

**Fig. 4.**
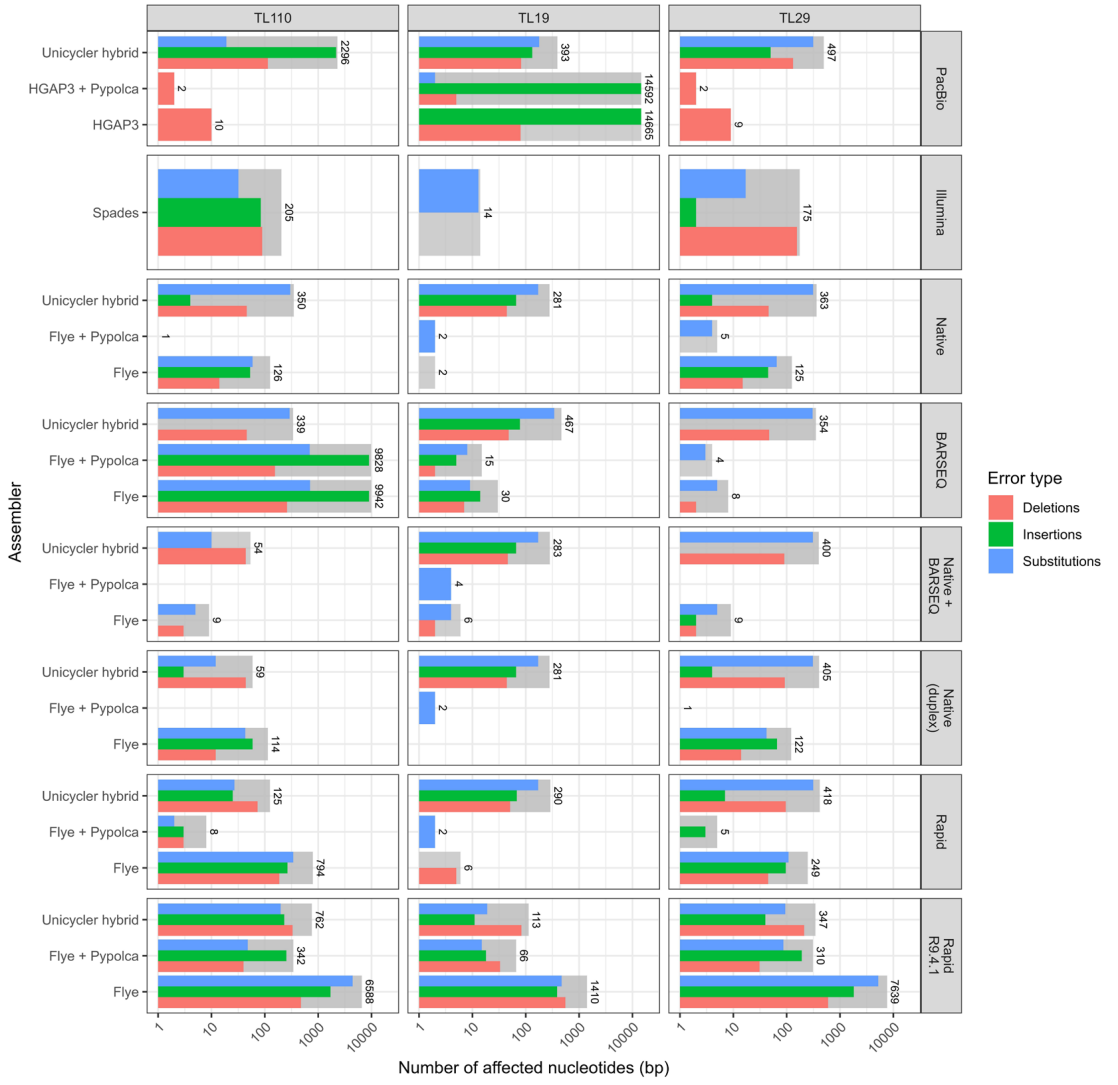
Variant calling including insertions, deletions and substitutions in the genomes of the TL110, TL19 and TL29 strains obtained using different sequencing and assembly strategies. Reference genomes were generated as described in the Methods section. Grey bars represent the sum of all the other variants. The values indicate the total number of nucleotide differences across full genomes (>2.5 Mbp).

Assemblies generated from all sequencing and assembly strategies were compared against the reference genomes to evaluate their accuracy (Figs 4 and S4). Assemblies were classified as ‘perfect’ if identical to the reference and ‘near-perfect’ if they had fewer than ten total single-nucleotide variants (SNVs) plus indels, with no large insertions or deletions [13] and matched the reference in the number of contigs (i.e. a single contig). More contigs can result in unreliable assemblies, even with a low number of SNVs and indels (e.g. TL29 BARSEQ in Figs2  4), given that it is impossible for the assemblers to correctly organize and reorient these contigs.

We first evaluated if Medaka polishing improved the R10.4.1 data assemblies generated with Flye. However, it did not consistently reduce errors across the different assemblies (Fig. S5), and thus, it was excluded from further analysis.

For single-technology assemblies, the Native data combined with BARSEQ strategy assembled with Flye were the only approach that produced ‘near-perfect’ assemblies for all strains. Combining reads from both library preparation strategies significantly improved the results obtained separately, except for TL19, where Native data alone achieved an assembly with only 2 bp differences (Fig. 4).

PacBio-only assemblies achieved ‘near-perfect’ status for the TL29 and TL110 strains. However, HGAP3 misassembled a region of the TL19 chromosome, causing a >9 600 bp duplication (Fig. S6), detected by the Medaka variant calling it a >14 500 bp insertion (Fig. 4). Excluding this misassembled region, there were a total of 80 bp differences, all of them deletions.

Assemblies using Native Duplex reads performed slightly better than those done with the Native (non-duplex) dataset. Notably, the assembly of TL19 with Duplex reads and Flye was the only single-technology strategy to achieve a perfect assembly, whereas assembly with Native (non-duplex) reads resulted in only 2 bp differences. Regarding TL110 and TL29, the total number of differences ranged from 114 to 126 bp. The error distribution was similar, but Duplex reads tended to increase the insertion rate and decrease the substitutions compared to regular Native reads.

The assemblies generated from the Native Barcoding kit outperformed those generated from the Rapid kit in all cases. While the latter achieved a ‘near-perfect’ assembly for TL19 with only 6 bp differences, it produced 249 and 794 bp differences for TL29 and TL110, respectively.

Illumina data assembled with SPAdes performed particularly well for the TL19 strain, with 14 bp differences, all substitutions. TL29 and TL110 presented 175 and 205 differences, respectively. On the other hand, BARSEQ assemblies performed the best for TL29 with only 8 bp differences, followed by TL19 with 30 bp differences. Interestingly, for the TL110 strain, the BARSEQ assembly obtained a total of 9942 differences, most of them insertions located in one region.

Finally, ONT Rapid sequencing with the R9.4.1 Flow cell yielded the least satisfactory results, with 1410, 6588 and 7639 bp differences for TL19, TL110 and TL29, respectively.

Long-read-first hybrid assemblies performed better than short-read-first assemblies in terms of accuracy except for the aforementioned large insertions in the TL110 BARSEQ and TL19 PacBio assemblies, which Illumina polishing could not resolve.

Polishing the assemblies with Illumina reads resulted in a diminution of the differences in all the cases, except for TL19 Duplex, in which the assembly was already perfect and where Pypolca introduced two substitutions. Notably, Illumina polishing produced perfect assemblies for TL110 Native Duplex, as well as TL110 and TL29 Native (non-duplex) combined with BARSEQ. Furthermore, several near-perfect assemblies were obtained after polishing with Pypolca, including assemblies initially containing up to 794 bp differences, such as TL110 Rapid. Finally, polishing the R9.4.1 assemblies with Illumina dramatically reduced the number of discrepancies (Fig. 4).

### Quality of the assembled genomes: coding sequences and proportion of unannotated genes

The assembled genomes of the *P. freudenreichii* strains were annotated and evaluated for their number and composition of coding sequences (CDS), as well as their proportion of unannotated genes (i.e. annotated as ‘hypothetical protein’) (Table 1, Figs 3 and S7), across the different sequencing and library preparation strategies.

Indels and SNVs can significantly impact gene annotation by causing frameshifts or altering reading frames. This effect is clearly demonstrated in the assembly generated with the Rapid kit for TL110 using Flye, where 798 bp differences in a 2.56 Mbp genome (99.97% similarity) resulted in a total number of CDS of 2520, 292 more than the reference, highlighting the need for accurate assembly to ensure reliable gene prediction.

The ONT Rapid method sequenced with R9.4.1 showed the most discrepancies in total CDS numbers compared to the reference genomes. Among the assemblers, Unicycler had the highest number of discrepancies. Nonetheless, most assemblies (35 out of 66) had fewer than ten CDS differences compared to the references (Table 1).

Regarding the proportion of unannotated genes, all sequencing and assembly strategies obtained similar values, generally ranging from 40 to 41% CDS annotated as hypothetical proteins. Illumina-only and TL110 ONT Rapid sequenced with R10.4.1 and assembled with Flye showed values from ~42–43.7 %, respectively. The R9.4.1 assemblies showed the highest proportion of unannotated genes. Polishing the latter with Illumina reads using Pypolca reduced these proportions to levels comparable to other strategies (Fig. 3).

Importantly, having comparable numbers of CDS and proportion of unannotated genes does not ensure similar CDS composition. This is evident in the Illumina-only assemblies, which, despite the low number of indels and SNV counts, showed the greatest discrepancies in the accessory gene content (i.e. genes that were not common for all the assemblies of a specific strain) compared to those in the reference, highlighting the importance of long-reads and contiguity for that purpose. In general, assemblies performed with SPAdes, Unicycler, as well as assemblies from BARSEQ and R9.4.1 data produced the most dissimilar results (Fig. S7).

### Comparison of detected methylation motifs between ONT and PacBio

The methylation profiles of the three *P. freudenreichii* strains were generated for the Native R10.4.1 data with MicrobeMod [46] and Nanomotif [45] and compared to the gold standard, PacBio. Three methylated motifs were detected for each of the strains, with the representation of all the methylation types present in bacteria (6mA, 5mC and 4mC) (Table 2). Both PacBio and ONT utilizing Nanomotif enabled the detection of reliable motifs, i.e. those methylated at or very close to 100% of the sites within the genome, whereas MicrobeMod detected motifs at lower percentages and failed to identify the type I partner motifs, i.e. the motifs on the complementary strands.

**Table 2.** Methylation motifs, types and occurrences in the genomes of the three *P. freudenreichii* strains obtained with PacBio and ONT R10.4.1

Strain	PacBio motif	Methylated sites/motif occurrence in the genome	Nanomotif motif	Methylated sites/motif occurrence in the genome	MicrobeMod motif	Methylated sites/motif occurrence in the genome
**TL110**	GA**A**NNNNNNNC**T**T	489/489 (100 %)	GA**A**NNNNNNNC**T**T	444/462 (96.10 %)	GA**A**NNNNNNNC**T**T†	224/235 (95.3 %)
	CC**A**NNNNNNNR**T**AY	749/749 (100 %)	CC**A**NNNNNNNR**T**AY	712/730 (97.53 %)	C**A**NSNNSNNN**T**A§	305/3526 (8.3 %)
	GR**GC**YC^*^	10 433/10 433 (100 %)	GR**GC**YC	10 341/10 623 (97.35 %)	GG**GC**CC	2866/3206 (89.4 %)
**TL29**	GA**A**NNNNNNNC**T**T	510/523 (97.5 %)	GA**A**NNNNNNNC**T**T	443/462 (95.89 %)	A**A**NNNNNNNN**T**T†	468/2283 (20.5 %)
	CC**A**NNNNNNNR**T**AY	519/522 (99.5 %)	CC**A**NNNNNNNR**T**AY	712/730 (97.53 %)	T**A**NNNSNNSN**T**GG†,§	258/1036 (24.9 %)
	GR**GC**YC*	11 142/21 054 (53 %)	GR**GC**YC	20 718/21 250 (97.50 %)	GG**GC**CC	2814/3147 (89.4 %)
**TL19**	GA**A**NNNNNNNC**T**T	458/458 (100 %)	GA**A**NNNNNNNC**T**T	445/460 (96.74 %)	A**A**NNNNNNNN**T**T†	480/2230 (21.5 %)
	C**A**GNNNNNR**T**TG	1021/1026 (99.6 %)	C**A**GNNNNNR**T**TG	1010/1020 (99.02 %)	CA**A**NNNSNNC**T**GS†,§	323/470 (68.7 %)
	TC**G**W**C**GA	3886/4170 (93.2 %)	TC**G**W**C**GA	3718/4059 (91.60 %)	Not detected‡	Not detected

Motifs correspond only to one of the strands and the total numbers are the sum of both the specified motif and its partner motif. ColoredColoured nucleotides represent the methylated bases. Methylation types correspond to 4mC, 5mC, and 6mA.

* Motif determined by homology.

†Motifs only detected for one strand.

‡MicrobeMod detected a total of 2100 4mC methylated sites, yet no corresponding motif was assigned.

§Motif corresponds to the complementary strand.

Despite encountering nucleotide modification hints in the raw data analysis, PacBio RSII does not allow for the detection of 5mC motifs. Thus, the 5mC methylated GRGCYC motifs in TL110 and TL29 were determined by the homology of the restriction-modification (RM) systems to other bacteria. Conversely, Nanomotif detected the motif GRGCYC in 97.5% of the total sites within the genome, whereas MicrobeMod determined that the methylated motif was GGGCCC, which was present in 89.4% of the total sites. To further investigate this matter, an assay utilizing restriction enzymes was conducted. ApaI, which recognizes GGGCCC, and BanII, targeting GRGCYC, were used to digest the native DNA of strains TL110, TL29 and TL19. Neither enzyme successfully digested the DNA of TL110 and TL29, indicating that the correct motif for these strains is GRGCYC (Fig. S8), which also encompasses GAGCTC, GAGCCC and GGGCTC and is not limited to GGGCCC since otherwise BanII would have digested other versions of the motif not protected by methylation.

Finally, PacBio and Nanomotif detected the 4mC palindromic motif TCGWCGA in TL19. Conversely, MicrobeMod was capable of determining the presence of 4mC methylation in the sample but did not manage to correctly assign a motif.

Overall, PacBio delivered the most reliable motifs, closely followed by Nanomotif, which additionally detected the GRGCYC 5mC motif. Methylated motif detection using MicrobeMod was less reliable and accurate. Nonetheless, MicrobeMod offers a feature to annotate RM systems and perform homology searches to determine the motifs utilizing PacBio data contained in REBASE [50] as a reference. This enabled the correct identification of the type II GRGCYC motif and all type I motifs (data not shown).

## Discussion

Despite advancements in the cost-effectiveness and throughput of DNA sequencing, obtaining complete, high-quality genomes remains challenging. Illumina platforms are known for generating accurate, low-cost short reads [9] but struggle with resolving complete circular genomes and long repeats due to their limited read length. PacBio generates high-quality, long reads for complete genome reconstruction [26], while ONT provides even longer reads at a lower cost, though with historically higher error rates [7,53, 54].

PacBio or hybrid assemblies combining Illumina and ONT data are the most commonly used approaches for recovering high-quality, complete genomes. While hybrid assemblies are more cost-effective, they require complex library preparation and data analysis. PacBio remains more straightforward but is expensive due to high equipment costs. Recent improvements in ONT technology, such as better chemistry and data analysis tools, have enhanced its data quality [14,55, 56] and enabled reliable methylation analysis [45]. However, many researchers still prefer other strategies due to established infrastructure or unclear guidance on optimal ONT library preparation.

We evaluated the performance of ONT using the latest R10.4.1 Flow cells and various sequencing strategies on three *P. freudenreichii* strains, which present challenges due to their high G+C content and extensive repeats [17,57, 58]. Previous ONT attempts with R9.4.1 Flow cells yielded unsatisfactory single-base accuracy, despite successful sequencing of other microbes (own internal data).

Illumina protocols are among the most popular, requiring low DNA concentrations and using PCR for enrichment. In contrast, third-generation platforms like ONT typically require higher DNA inputs, which can be challenging. ONT offers optional enrichment protocols such as transposase shearing with PCR (Rapid PCR kit) or multiple displacement amplification. However, in our experience, building DNA libraries with the Rapid PCR kit was often unsuccessful and appeared to be highly strain-dependent. To address this, we have developed a custom enrichment and barcoding protocol, BARSEQ, which allows low-input DNA preparation and is 20 times more cost-effective than commercial solutions (Table S1).

Comparing sequencing costs across platforms is difficult due to differences in throughput, coverage and multiplexing. Nonetheless, we estimate sequencing an average bacterial genome (2.5M) with ONT costs around $18 (for 96 isolates on PromethION with the Native Barcoding kit), whereas PacBio costs are eight to ten times more expensive. While PacBio’s Revio system may narrow this cost gap, ONT’s ~$1000 MinION remains the most affordable option.

While BARSEQ has its advantages, sequencing libraries without enrichment with ONT offers key benefits, such as generating very long reads and allowing simultaneous analysis for basecalling and DNA modifications, such as methylation. Although bacterial DNA modification calling is still maturing, our data show that methylome recovery is already feasible using the latest basecaller (Dorado v0.7) and methylation calling tools, such as MicrobeMod [46], and particularly Nanomotif [45], at least for the studied *P. freudenreichii* strains. Libraries prepared without PCR also avoid errors associated with primer bias, polymerase errors and transposase-related errors in regions with high or very low G+C content [59,61]. Conversely, native DNA often contains modifications, which can lead to basecalling errors [8]; achieving the best base-to-base quality on ONT platforms may require the removal of these modifications with PCR.

Our data indicate that ONT’s R10.4.1 Flow cells combined with V14 chemistry can recover very high-quality, complete bacterial genomes. BARSEQ provides a viable option for low DNA concentration samples but falls short in achieving the N50 length needed for complete genome recovery. The highest-quality assemblies, comparable to those obtained with PacBio or long-read-first hybrid assemblies, are achieved by combining long reads from the Native protocol with BARSEQ. This improvement is likely due to the correction of systematic errors inherent in each preparation strategy when data from both approaches are merged.

Another benefit of using combined data from Native and BARSEQ protocols is that the same low-cost platform can generate all the data necessary for the recovery of high-quality, complete genomes. Other authors have shown that perfect or near-perfect assemblies from ONT Native data alone can be recovered with Trycycler [62]. In our case, Trycycler reference assemblies introduced only one change when polishing ONT data with Illumina reads. However, Trycycler is time-consuming and requires advanced bioinformatics capabilities and computational resources [13,30]. In general, when perfect or near-perfect assemblies are not required, Flye assembly of ONT R10.4.1 data obtained from a single wet-lab protocol yields very good results, especially with the Native kit, sometimes even achieving perfect or near-perfect assemblies, as shown in this study. It is reasonable to assume that genomes considered less challenging to sequence (e.g. those with even G+C content, low proportions of repeated regions or low methylation levels) will yield high-quality genomes without requiring a combination of different data types.

We have observed a better performance of the ONT Native Barcoding kit compared to that of the Rapid kit. It is important to mention that Native kits (ligation) typically generate more data than Rapid protocol, which can influence coverage and therefore quality of assembly. Moreover, the Rapid kit, as well as Illumina’s Nextera kit, relies on transposases, which have been reported to affect genomic and metagenomic reconstructions due to their higher affinity for G+C-rich regions [63].

Finally, while the duplex reads in this study contributed to 17–20% of the data with Q25, surpassing the ~2–6% reported in other studies [56], these results are still far from the ~40% reported by ONT. We observed a considerable increase in read quality when extracting duplex reads, which improved assembly compared to data derived from the Native kit. Although other studies [56,62] have reported superior results with Duplex data over non-duplex Native data, we found that the final assemblies produced by both methods were very similar.

Our results align with previous studies [13,14, 55] showing that long-read-first hybrid assemblies outperform short-read-first approaches in contiguity and accuracy. Despite this, short-read-first hybrid assembly remains the gold standard for plasmid detection [13,64]. However, since the *P. freudenreichii* strains studied here are plasmid free, we could not assess their performance in this regard.

The main limitations of this study include the lack of multiple technical replicates, which may limit the generalizability of the findings. Additionally, the study tested a limited range of microorganisms, which could affect the applicability of the results to other species. Finally, the absence of plasmids in the tested samples precludes the evaluation of sequencing performance on extrachromosomal elements.

In conclusion, highly comparable, high-quality complete bacterial genomes can be reconstructed using ONT with R10.4.1 Flow cells and V14 chemistry. The highest-quality genomes were reconstructed by two possible approaches: (i) long-read-first hybrid assembly combining data from two sequencing strategies, i.e. ONT/PacBio long reads polished with Illumina data, or (ii) using single-platform-generated data from ONT Native Barcoding complemented with PCR-based BARSEQ. Taking into consideration the significantly lower costs related to the sequencing platform, library preparation, Flow cells and throughput, it appears that ONT alone is the most optimal sequencing strategy for this task.

## supplementary material

10.1099/mgen.0.001316 Uncited Supplementary Material 1.

## Abbreviations

BARSEQ: barcode-amplified random sequencing
CDS: coding sequences
NCBI: National Center for Biotechnology Information
ONT: Oxford Nanopore Technologies
PacBio: Pacific Biosciences
RM: restriction-modification
SNV: single nucleotide variant
YEL: yeast extract lactate
