# Supplementary material for: Matching excellence: Oxford Nanopore Technologies’ rise to parity with Pacific Biosciences in genome reconstruction of non-model bacterium with high G+C content
Source: Microb Genom. 2024 Nov 11;10(11):001316. doi: 10.1099/mgen.0.001316 (PMC11649196; doi:10.1099/mgen.0.001316)
Supplement: Uncited Supplementary Material 1. [file mgen-10-01316-s001.pdf]

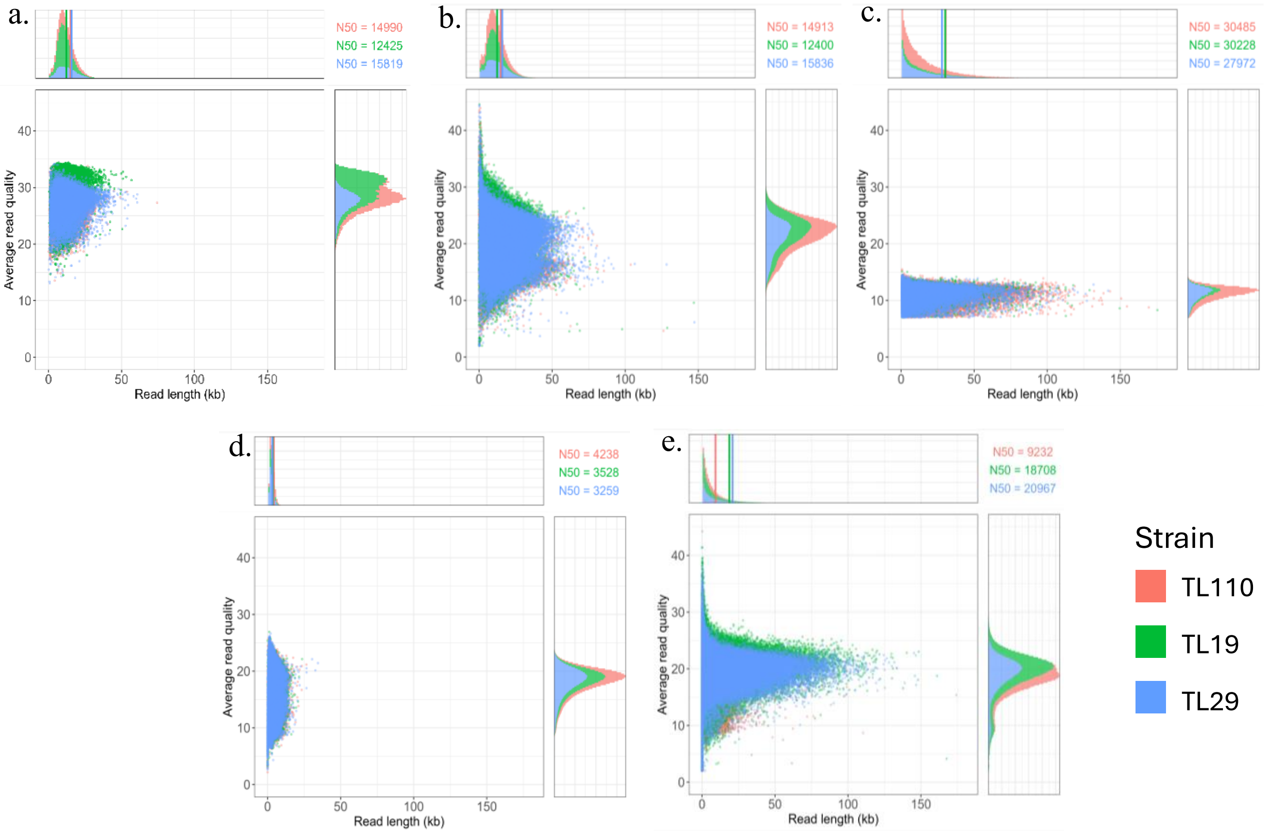

Fig. S1. Quality biplots for ONT data. (a) Native “duplex” R10.4.1; (b) Native R10.4.1; (c) Rapid R.9.4.1; (d) BARSEQ; (e) Rapid R10.4.1.

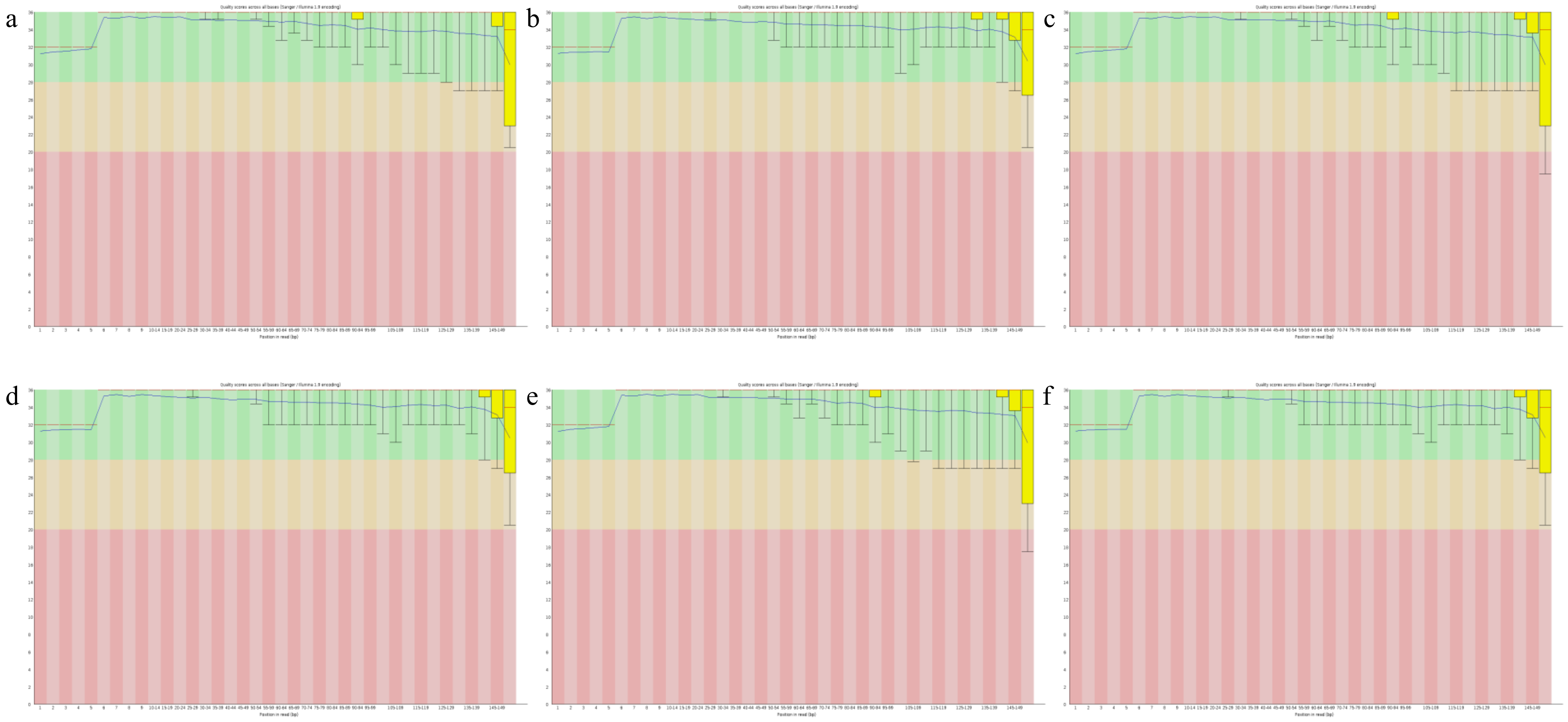

Fig. S2. Quality scores for the Illumina reads (a) TL110 R1; (b) TL110 R2; (c) TL29 R1; (d) TL29 R2; (e) TL19 R1; (f) TL19 R2.

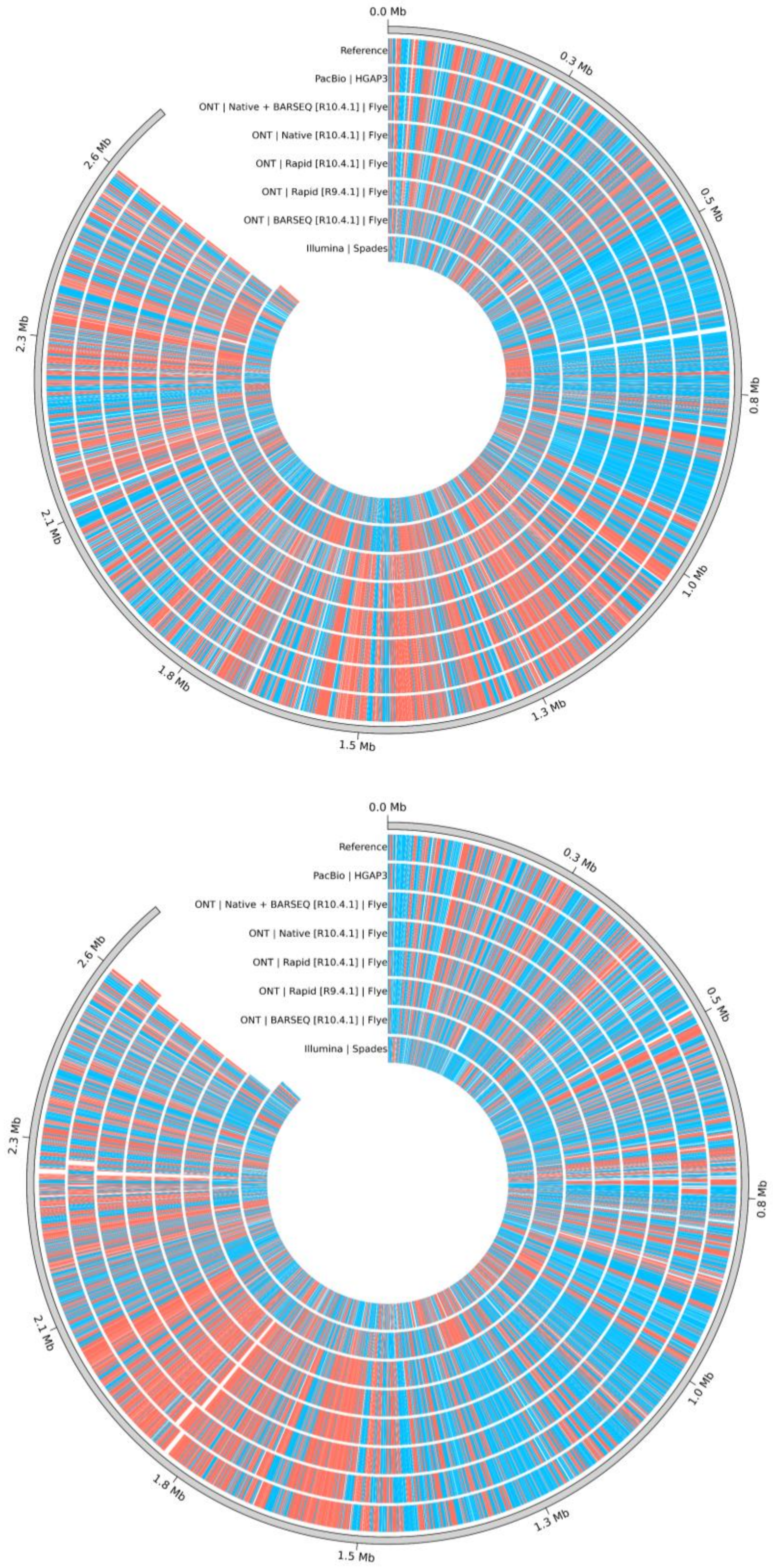

Fig. S3. Circularized genome assemblies obtained from a single sequencing technology (ONT, PacBio, or Illumina) for the TL110 (top) and TL19 (bottom) strains. The reference assembly is placed in the outermost ring. Red and blue stripes correspond to coding sequences (CDS) in the forward and reverse strands, respectively.

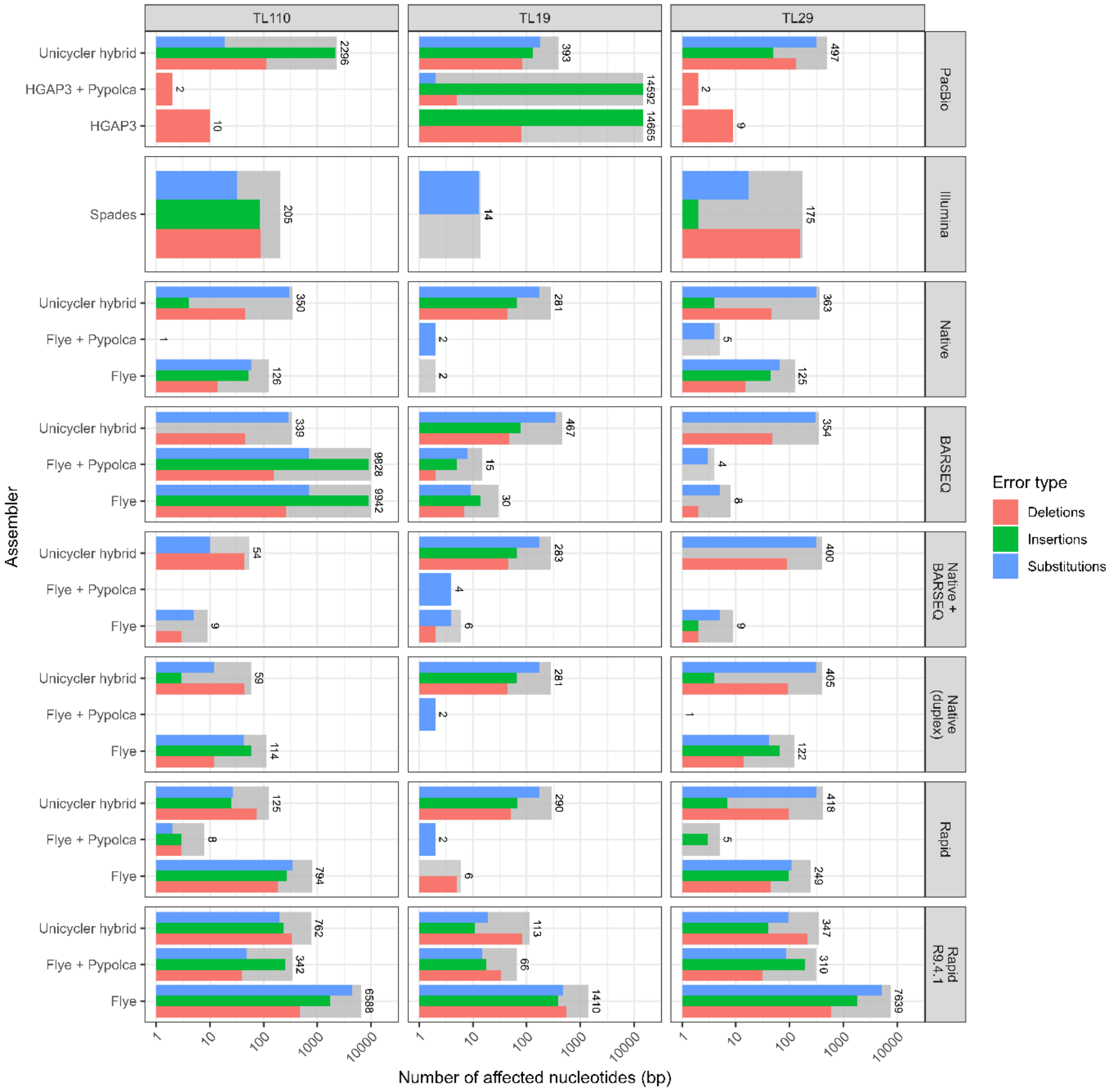

Fig. S4. Substitution types in the genomes of the TL110, TL19, and TL29 strains obtained using different sequencing and assembly strategies. Reference genomes were generated as described in the Materials and Methods section. Grey bars represent the sum of all the other variants. The values indicate the total number of nucleotide differences across full genomes (> 2.5 Mbp).

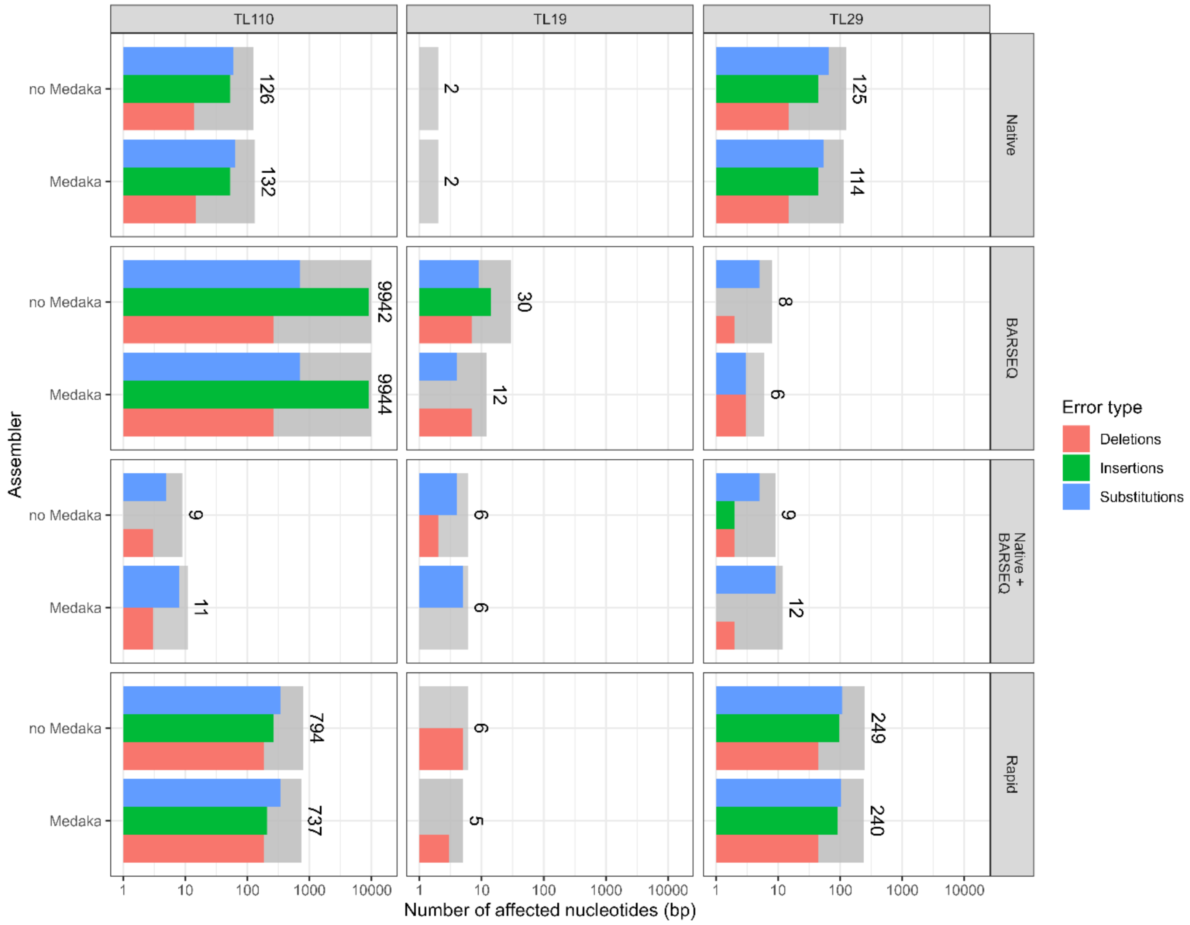

Fig. S5. Effect of Medaka polishing in the variant calling including insertions, deletions, and substitutions in the genomes of the TL110, TL19, and TL29 strains obtained using different library preparation strategies, sequenced with R10.4.1 Flow cells, and assembled with Flye. Reference genomes were generated as described in the Materials and Methods section. Grey bars represent the sum of all the other variants. The values indicate the total number of nucleotide differences across full genomes (> 2.5 Mbp).

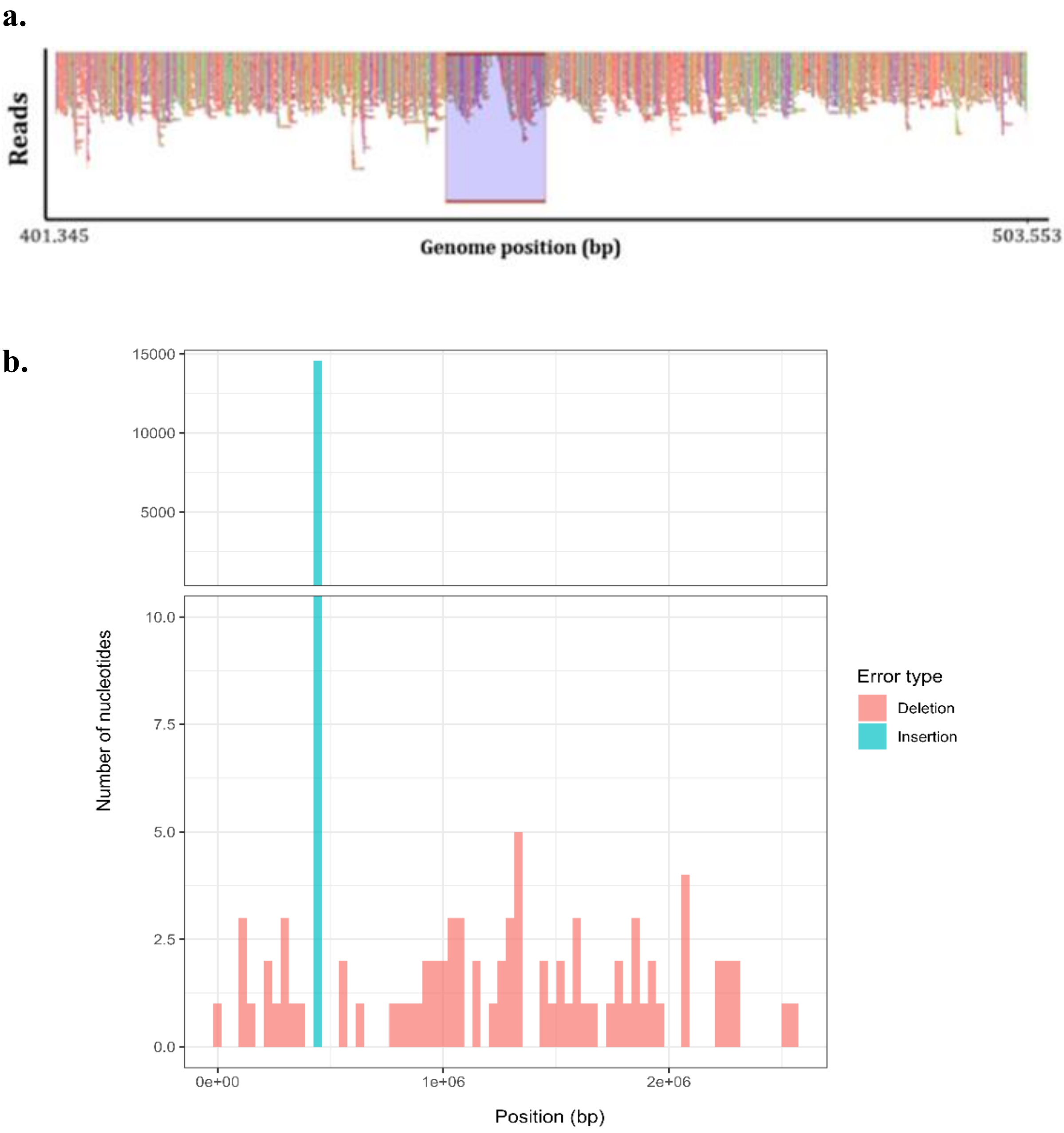

Fig. S6. Misassembly in a specific region (44024 – 464453 bp) in the genome of *P. freudenreichii* TL19 sequenced with Pacbio RSII and assembled with HGAP3. a) PacBio reads alignment showing the lack of coverage in the aforementioned region, which resulted in a duplication of > 9600 bp. b) Histogram plot showing the number and distribution of indels and substitutions when comparing the assembly against the reference generated as described in the Materials and Methods section.

a.

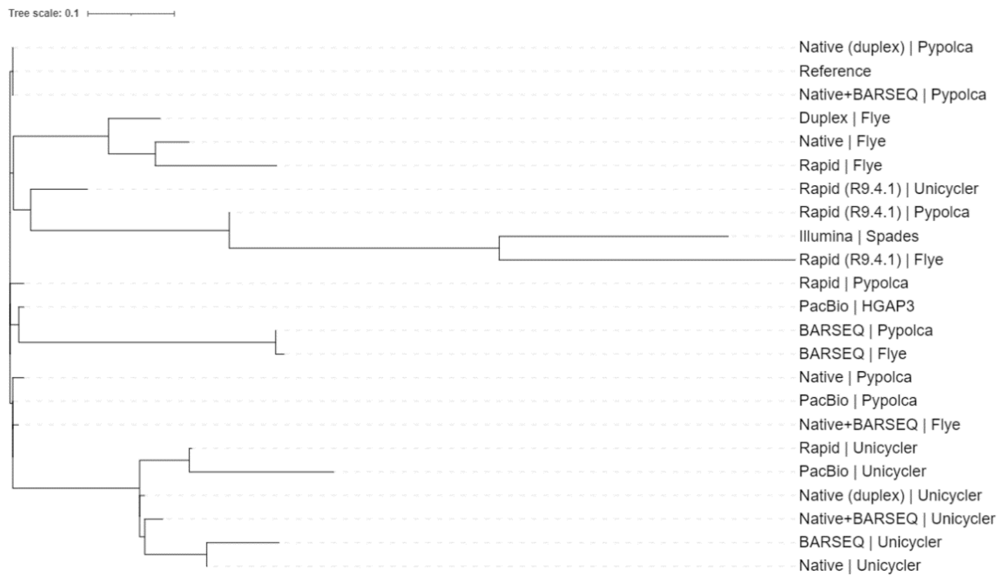

b.

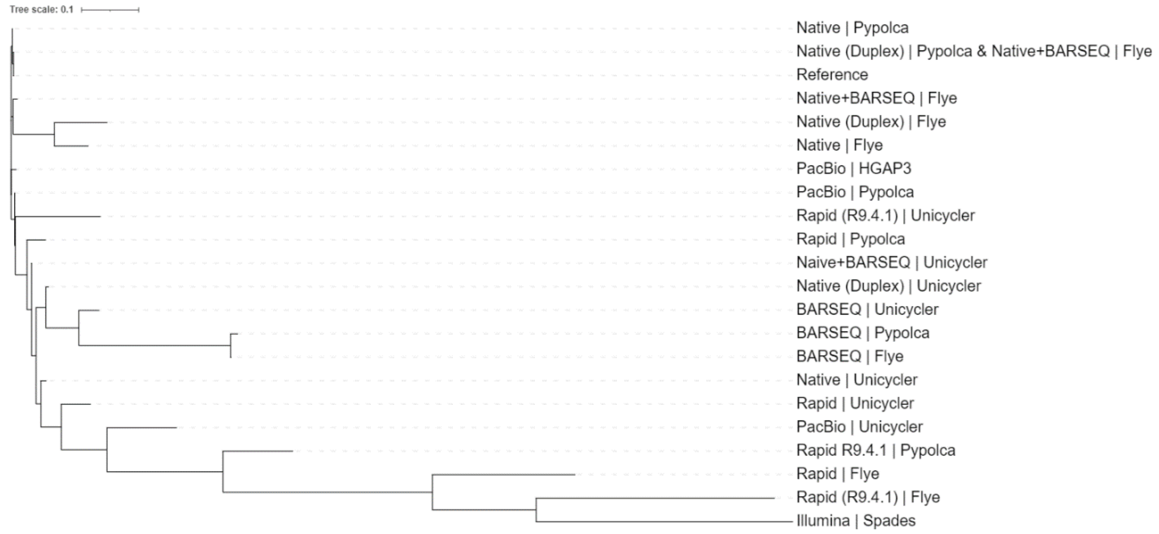

c.

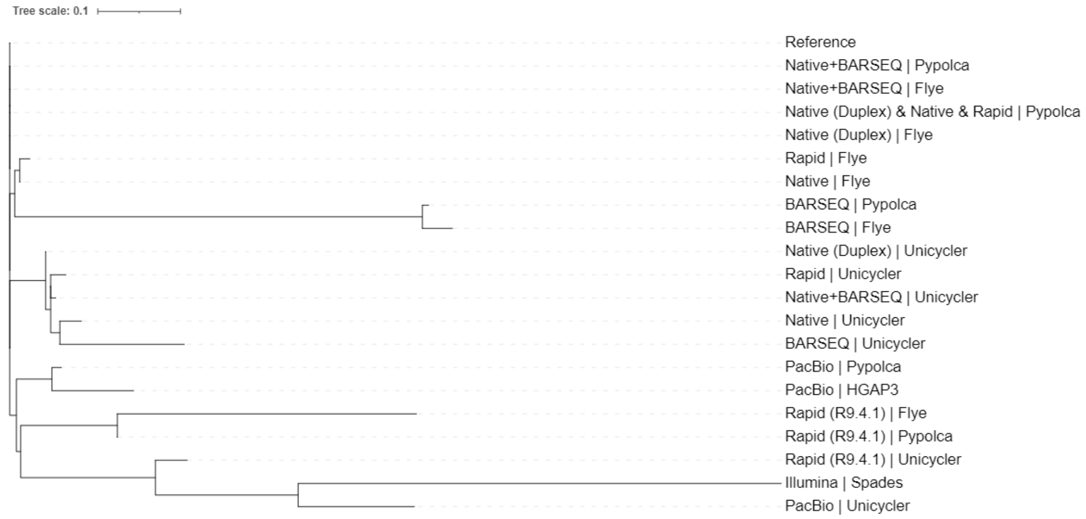

Fig. S7. Similarity tree based on accessory binary genes after pangenome analysis for (a) TL29 (b) TL110 and (c) TL19. The assembler employed is indicated after the sequencing strategy. Unicycler and Pypolca correspond to short-read-first and long-read-first hybrid assemblies, respectively.

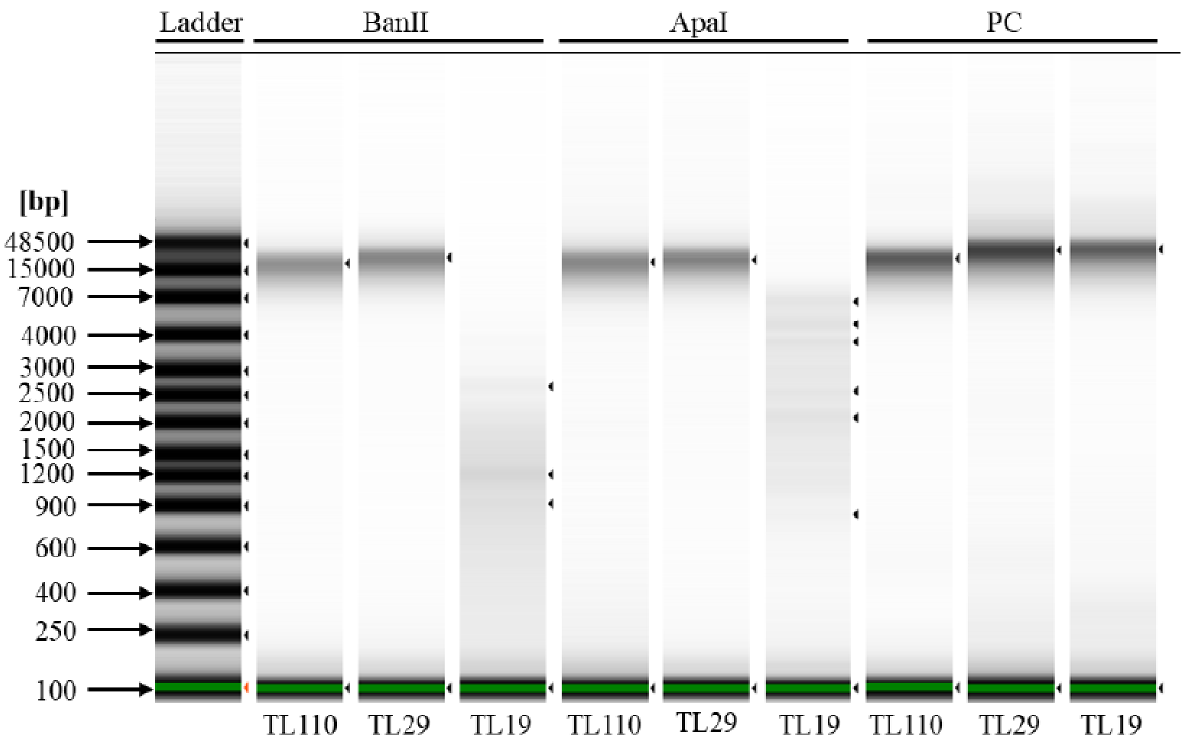

Figure S8. Methylation motifs discrimination using restriction enzymes assay. The TapeStation 4200 results showing the native genomic DNA of three *P. freudenreichii* strains (TL29, TL110 and TL19) treated with ApaI and BanII restriction enzymes in the presence of non-digested DNA positive control (PC). The assay demonstrates the presence of the methylated motif GRGCYC in strains TL110 and TL29 as it provides protection from both restriction enzymes, ApaI that recognizes GGGCCC and BanII targeting GRGCYC. In conclusion, ONT failed to identify the correct motif and PacBio relied on homology to find the correct motif.

Table S1. Comparison of costs and time for DNA library preparation between low and medium throughput platforms by ONT, Illumina and PacBio

	RAPID	NATIVE	BARSEQ	Illumina	PacBio
Platform cost ($)	~1,000-9,000 MinION or P2Solo	~1,000-9,000 MinION or P2Solo	~1,000-9,000 MinION or P2Solo	~125,000 MiSeq	~400,000 Sequel II
Library preparation kit ($)	990	799	900	3,400	995
Flow cell cost ($)	500-1,000	500-1,000	500-1,000	750-1,000	1,500-2,000
Runtime (h)	48-72	48-72	48-72	39	24-30
Library prep (min)	30	180	240	90	180
Estimated output per run (Gb)	50-290*	50-290*	50-290*	7.5-8.5	30-90
Read length (Kbp)	3-20Kb	20-100Kb	3-4Kbp	0.25Kbp	15-20Kbp

The information about prices given in USD is compiled from official web pages and price requests from vendors. The prices provided are approximate and may vary depending on personalized agreements. *The throughput for ONT is given for a single MinION flow cell (up to 50 Gb) or a single PromethION flow cell (up to 290 Gb).

Table S2. Adapter and barcodes sequences

Name	Sequence (5'-3')
BRK01	GTCTCGTCCGCTCGGCACAAAGACACCGACAACTTTCTTGTCTCGTGGGCTCGG
BRK02	GTTAGTTGATGTAGTACAGACGACTACAAACGGAATCGAGTCTCGTGGGCTCGG
BRK03	GTCTCGTCCGCTCGGCCTGGTAACTGGGACACAAGACTCGTCTCGTGGGCTCGG
BRK04	GTTAGTTGATGTAGTTAGGGAAACACGATAGAATCCGAAGTCTCGTGGGCTCGG
BRK05	GTCTCGTCCGCTCGGAAGGTTACACAAACCCTGGACAAGGTCTCGTGGGCTCGG
BRK06	GTTAGTTGATGTAGTGACTACTTTCTGCCTTTGCGAGAAGTCTCGTGGGCTCGG
BRK07	GATATGATATAGATAAAGGATTCATTCCCACGGTAACACGTCTCGTGGGCTCGG
BRK08	GTTAGTTGATGTAGTACGTAACTTGGTTTGTTCCCTGAAGTCTCGTGGGCTCGG
BRK09	GTCTCGTCCGCTCGGAACCAAGACTCGCTGTGCCTAGTTGTCTCGTGGGCTCGG
BRK10	GTTAGTTGATGTAGTGAGAGGACAAAGGTTTCAACGCTTGTCTCGTGGGCTCGG
BRK11	GTTAGTTGATGTAGTTCCATTCCCTCCGATAGATGAAACGTCTCGTGGGCTCGG
BRK12	GTCTCGTCCGCTCGGTCCGATTCTGCTTCTTTCTACCTGGTCTCGTGGGCTCGG
BRK13	GTCTCGTCCGCTCGGTCACACGAGTATGGAAGTCGTTCTGTCTCGTGGGCTCGG
BRK14	TACATTGATGCATGGTCTATGGGTCCCAAGAGACTCGTTGTCTCGTGGGCTCGG
BRK15	GTTAGTTGATGTAGTCAGTGGTGTTAGCGAGGTAGACCTGTCTCGTGGGCTCGG
BRK16	TACATTGATGCATGGAGTACGAACCACTGTCAGTTGACGGTCTCGTGGGCTCGG
BRK17	GTCTCGTCCGCTCGGATCAGAGGTACTTTCCTGGAGGGTGTCTCGTGGGCTCGG
BRK18	GTTAGTTGATGTAGTGCCTATCTAGGTTGTTGGGTTTGGGTCTCGTGGGCTCGG
BRK19	GTTAGTTGATGTAGTATCTCTTGACACTGCACGAGGAACGTCTCGTGGGCTCGG
BRK20	GTTAGTTGATGTAGTATGAGTTCTCGTAACAGGACGCAAGTCTCGTGGGCTCGG
BRK21	GTTAGTTGATGTAGTTAGAGAACGGACAATGAGAGGCTCGTCTCGTGGGCTCGG
BRK22	GTTAGTTGATGTAGTCGTACTTTGATACATGGCAGTGGTGTCTCGTGGGCTCGG
BRK23	GTCTCGTCCGCTCGGCGAGGAGGTTCACTGGGTAGTAAGGTCTCGTGGGCTCGG
BRK24	GTTAGTTGATGTAGTCTAACCCATCATGCAGAACTATGCGTCTCGTGGGCTCGG
BRK25	GTCTCGTCCGCTCGGCATTGCGTTGCATACCCAACTTACGTCTCGTGGGCTCGG
BRK26	TACATTGATGCATGGATGAGAATGCGTAGTCGCTGTATGGTCTCGTGGGCTCGG
BRK27	GTCTCGTCCGCTCGGTGTAAGAGGTGAATCTAACCGTCGGTCTCGTGGGCTCGG
BRK28	GTTAGTTGATGTAGTGATACGGTGCCTTCTTAGGTTTCAGTCTCGTGGGCTCGG
BRK29	GTTAGTTGATGTAGTGGTCTGTCAACCCAAGGTGTCTAGGTCTCGTGGGCTCGG
BRK30	GTTAGTTGATGTAGTTGGGTCGAAGTAGATCCTCACTGAGTCTCGTGGGCTCGG
BRK31	GTCTCGTCCGCTCGGCAATGTAACTGATTGCTGTACGCAGTCTCGTGGGCTCGG
BRK32	GTTAGTTGATGTAGTATGACGTTGTCGGACTTCTACTGGGTCTCGTGGGCTCGG
BRK33	GTCTCGTCCGCTCGGAGTTACCCAACCGTACCAAGTCTGGTCTCGTGGGCTCGG
BRK34	GTTAGTTGATGTAGTGCCTTTGACTTGAGTTCTTCGTCCGTCTCGTGGGCTCGG

BRK35	GTCTCGTCCGCTCGGGCAGTCCCTCAGCTTCGTAAGTAGGTCTCGTGGGCTCGG
BRK36	GTTAGTTGATGTAGTTGTTTCCTCCTCTAACTGGGACATGTCTCGTGGGCTCGG
BRK37	GTCTCGTCCGCTCGGTGATACTAAGCATCAATCGCAAGCGTCTCGTGGGCTCGG
BRK38	GTTAGTTGATGTAGTTTCTCTGTATCGTCCTCCTGTGGTGTCTCGTGGGCTCGG
BRK39	GTTAGTTGATGTAGTGAGAGGCTCTAGTTGACACTGTGGGTCTCGTGGGCTCGG
BRK40	GTTAGTTGATGTAGTGGCTATCCTTGGTCATCCAAACTAGTCTCGTGGGCTCGG
BRK41	GTTAGTTGATGTAGTCGTGTACTTCTCTGGACGAACTCCGTCTCGTGGGCTCGG
BRK42	GTCTCGTCCGCTCGGCTGGCAGGTATGCCTTACACGTAGGTCTCGTGGGCTCGG
BRK43	GTTAGTTGATGTAGTCTACCGTCGAGTCAACAACGAAAGGTCTCGTGGGCTCGG
BRK44	GTTAGTTGATGTAGTGAGTGGGAAGGAACCCTTTCTACTGTCTCGTGGGCTCGG
BRK45	GTCTCGTCCGCTCGGCACTGAAGGCATCTCTGTTGGATCGTCTCGTGGGCTCGG
BRK46	GTTAGTTGATGTAGTCAGGAGAATGAAGTGGAACACAGCGTCTCGTGGGCTCGG
BRK47	GTCTCGTCCGCTCGGGAACTACCTGTGGGAAAGTTGCACGTCTCGTGGGCTCGG
BRK48	GTTAGTTGATGTAGTTACAGGTGTACCACGTTCCAGATGGTCTCGTGGGCTCGG
BRK49	GTCTCGTCCGCTCGGCTAGATGTTCAAAGCTGCACCAGTGTCTCGTGGGCTCGG
BRK50	GTTAGTTGATGTAGTACGCAGGAAGTTACCAAAGTCCATGTCTCGTGGGCTCGG
BRK51	GTCTCGTCCGCTCGGGAGGACCCAGTAGGCTCATTCAACGTCTCGTGGGCTCGG
BRK52	TACATTGATGCATGGGTCCACGAACAATCTTGTCTCTCAGTCTCGTGGGCTCGG
BRK53	GTCTCGTCCGCTCGGCTTTGCATGAGACGGTCTGAATCTGTCTCGTGGGCTCGG
BRK54	GTTAGTTGATGTAGTCATGCTCCTTAGTCAAAGCTCTTGGTCTCGTGGGCTCGG
BRK55	GTCTCGTCCGCTCGGCGTAGATCAGGGTCTCATCTTCCAGTCTCGTGGGCTCGG
BRK56	GTCTCGTCCGCTCGGTTCATGCCACCTGTTGAGTAGTGAGTCTCGTGGGCTCGG
BRK57	TACATTGATGCATGGACTTCCGAAGGAGATTGACCTAGCGTCTCGTGGGCTCGG
BRK58	GTTAGTTGATGTAGTTCAGACTCACGGAGGAGTAACCTGGTCTCGTGGGCTCGG
BRK59	GTTAGTTGATGTAGTACCTTGCTTTCCCTTCTTGATTGAGTCTCGTGGGCTCGG
BRK60	GTTAGTTGATGTAGTCCATAGAAGCCTTGGTTGAACATGGTCTCGTGGGCTCGG
BRK61	TACATTGATGCATGGGTGCTGAGGCACATAGTACCCTCTGTCTCGTGGGCTCGG
BRK62	GTTAGTTGATGTAGTTACGTCCTGAAGTAAGTGTGGGTGGTCTCGTGGGCTCGG
BRK63	GTTAGTTGATGTAGTGTTCAAGACCCAGGAACTTCAGAAGTCTCGTGGGCTCGG
BRK64	GTTAGTTGATGTAGTGAAAGTCGATGAACGGTGTCTGTCGTCTCGTGGGCTCGG
BRK65	GTCTCGTCCGCTCGGCCTTGTCTGGAGGAAGACTGAGAAGTCTCGTGGGCTCGG
BRK66	GTCTCGTCCGCTCGGGAAGTTAGAAGCCACAAGGATCGGGTCTCGTGGGCTCGG
BRK67	TACATTGATGCATGGGGTGAGCACACGAGTATGACAAACGTCTCGTGGGCTCGG
BRK68	GTCTCGTCCGCTCGGCCACCTTCGTGTTTGCTTAGATTCGTCTCGTGGGCTCGG
BRK69	GTTAGTTGATGTAGTAGATCACATGAGGCTCGGACTGTAGTCTCGTGGGCTCGG
BRK70	GTTAGTTGATGTAGTACACTCCATTCGTAGGATCTCGGTGTCTCGTGGGCTCGG

BRK71	GTCTCGTCCGCTCGGCTGTTACTACCTGATGCTCCCAGGGTCTCGTGGGCTCGG
BRK72	GTTAGTTGATGTAGTGTCGGTATGGAAGACAGTCAGCTAGTCTCGTGGGCTCGG
BRK73	GTCTCGTCCGCTCGGGAGGGTTCTGTCATCCTGTTTCTTGTCTCGTGGGCTCGG
BRK74	GTTAGTTGATGTAGTAGTGGAAGTGTTGGGATGCTTGTAGTCTCGTGGGCTCGG
BRK75	GTCTCGTCCGCTCGGACAACAGGGTTCATCACAATGGTCGTCTCGTGGGCTCGG
BRK76	GTTAGTTGATGTAGTGTCCAGGGTTGATGTAACAAGCATGTCTCGTGGGCTCGG
BRK77	GTCTCGTCCGCTCGGGTTGTATCCCTGAGAAACAGGTCGGTCTCGTGGGCTCGG
BRK78	GTTAGTTGATGTAGTTTCTGATTCAAAGGTTCGGTTGTTGTCTCGTGGGCTCGG
BRK79	GTCTCGTCCGCTCGGCAGCAGTGAGAACTATCTCCGAGAGTCTCGTGGGCTCGG
BRK80	GTTAGTTGATGTAGTGAATCGCTATCCTATGTTCATCCGGTCTCGTGGGCTCGG
BRK81	GTCTCGTCCGCTCGGCCGAAACAACTTCACAAGATGAGGGTCTCGTGGGCTCGG
BRK82	GTTAGTTGATGTAGTTAGTCCTGGAACTCGACATACCGTGTCTCGTGGGCTCGG
BRK83	GTCTCGTCCGCTCGGTTCGACCTTACCTAGATCAAGCCAGTCTCGTGGGCTCGG
BRK84	GTTAGTTGATGTAGTTGGCACAGGTTCTAGGTCCACTACGTCTCGTGGGCTCGG
BRK85	GTCTCGTCCGCTCGGGATCATCCAACTAACTCCTCCGTTGTCTCGTGGGCTCGG
BRK86	GTCTCGTCCGCTCGGTACTTACGCTTGTTGGGATCACCTGTCTCGTGGGCTCGG
BRK87	GTCTCGTCCGCTCGGCCTCCCTAACAACAGGAGCATGTAGTCTCGTGGGCTCGG
BRK88	GTTAGTTGATGTAGTCTGCTTCGGATCGGTAGTAGAAGAGTCTCGTGGGCTCGG
BRK89	GTCTCGTCCGCTCGGCAACTAGCCAAACATTGATGCTGTGTCTCGTGGGCTCGG
BRK90	GTTAGTTGATGTAGTGCCTCAAACCGTACCCTCTACATCGTCTCGTGGGCTCGG
BRK91	TACATTGATGCATGGAGTAGCGTGAGTTCCTATGGAGCCGTCTCGTGGGCTCGG
BRK92	GTCTCGTCCGCTCGGGGTCCTGTATCTTTCCACTCACAAGTCTCGTGGGCTCGG
BRK93	GTCTCGTCCGCTCGGCCCAAGTCTGAAGTGATGGAAACTGTCTCGTGGGCTCGG
BRK94	GTTAGTTGATGTAGTGTAGGTGGCAGTTTGAGGACAATCGTCTCGTGGGCTCGG
BRK95	GTCTCGTCCGCTCGGAAGTCCATTCTTCTTCCAGACAGGGTCTCGTGGGCTCGG
BRK96	TACATTGATGCATGGATGGTGGACTCTATGACCGTTCAGGTCTCGTGGGCTCGG

Table S3. Selection of reference genomes. The candidate reference genomes considered were Assembly 1: ONT-derived assembly (ONT Native+BARSEQ data assembled with Trycycler + Illumina polishing with both Pypolca and Polypolish); Assembly 2: PacBio-derived assembly (PacBio RSII data assembled with Trycycler + Illumina polishing with both Pypolca and Polypolish). The mismatches between the assemblies identified with mappy were manually checked and, wherever possible, an informed decision was made as to which variant is more likely to be correct. To this end, annotations with Prokka were checked for context, including gene orientation, size, completeness and whenever appropriate, amino acid sequences of the products. When needed, the gene regions and/or amino acid sequences of the products were compared against other *P. freudenreichii* genomes available at NCBI with the assumption that since the data there originates from various sequencing platforms, the consensus values can be considered a real observation. All of the 16 mismatches were deletions in Assembly 2, which is therefore not particularly surprising that 14 of those were deemed incorrect, one was deemed incorrect in Assembly 1, and for one no conclusion could be made. It was somewhat surprising that several of the mismatches were found in corresponding regions in different strains, such as in 16S rRNA gene for all of the strains, one gene coding for a hypothetical protein and one intergenic region in two of the strains. It is worth noting that the observed differences could be biological in nature and in fact correct, however we have no way of determining this at this time. As a result, we decided to choose Assembly 1 as reference assembly for all of the strains.

Strain	Mismatch No.	Mismatch context	Context	Note	Presumed correct
TL110	1	Assembly 1: 676229-676259: GTGGCCCAACCTTTTGGGGGGGAGCCGTCGA<br>Assembly 2: 676229-676258: GTGGCCCAACCTTTT-GGGGGGAGCCGTCGA<br>*	16S ribosomal RNA<br>16S ribosomal RNA	Two variants of 16S rRNA gene exist in *P. freudenreichii*. The Assembly 1 version corresponds to what is found in all other strains at NCBI.	1
	2	Assembly 1: 1862384-1862414: GCGCGCAACGCCCAGCCCCCCCCCCGCACAC<br>Assembly 2: 1862382-1862411: GCGCGCAACGCCCAG-CCCCCCCCCGCACAC<br>*	Hypothetical protein<br>Hypothetical protein	Missing C in Assembly 2 moves the start codon and results in a product truncated at N-terminus.	1
	3	Assembly 1: 2010527-2010557: TACTGACACGCACCACCCCCCCCCACGCACC<br>Assembly 2: 2010524-2010553: TACTGACACGCACCA-CCCCCCCCACGCACC<br>*	Intergenic region<br>Intergenic region	Difference introduced to Assembly 2 by polishing (as compared to the original HGAP3 assembly).	1
TL19	1	Assembly 1: 187482-187512: CCTGAATGGCCTGCTGGGGGGTGTCCCACTC<br>Assembly 2: 187482-187511: CCTGAATGGCCTGCT-GGGGGTGTCCCACTC<br>*	IS3 family ISPfr11 transposase ORF B<br>IS3 family transposase ORF B	Change in number of Gs changes the translated sequence after 261 aa. The Assembly 1 version is widely represented in other *P. freudenreichii* strains.	1
	2	Assembly 1: 284612-284642: CTGTCCCTCCGGAATCCCCCCGGGCCCCTCC<br>Assembly 2: 284611-284640: CTGTCCCTCCGGAAT-CCCCCGGGCCCCTCC<br>*	Hypothetical protein<br>Hypothetical protein	Change in number of Cs changes the translated sequence after 226 aa. Only the Assembly 1 version is found in other *P. freudenreichii* strains.	1
	3	Assembly 1: 1503908-1503938: CGGGTGGGGGGTGTTGGGGGGATTTCCCGAA<br>Assembly 2: 1503906-1503935: CGGGTGGGGGGTGTT-GGGGGATTTCCCGAA<br>*	Intergenic region<br>Intergenic region	Region upstream of Glucan phosphorylase. Assembly 1 variant conserved among several *P. freudenreichii* genomes, introduced into Assembly 2 by polishing.	1
	4	Assembly 1: 1799393-1799423: GAAATCCCCCCAACACCCCCCACCCGAACCC<br>Assembly 2: 1799390-1799419: GAAATCCCCCCAACA-CCCCCACCCGAACCC<br>*	Intergenic region<br>Intergenic region	Region downstream of a Ketol-acid reductoisomerase. Assembly 1 variant conserved among *P. freudenreichii* genomes, introduced into Assembly 2 by polishing.	1
	5	Assembly 1: 1839889-1839919: TTCGCCACTCGTGTACCCCCCGAAAGGGGCC<br>Assembly 2: 1839884-1839913: TTCGCCACTCGTGTA-CCCCCGAAAGGGGCC<br>*	16S ribosomal RNA<br>16S ribosomal RNA	In many other *P. freudenreichii* genomes there is one or even two Cs fewer that in the Assembly 1.	2
TL29	1	Assembly 1: 266170-266200: GGTCGGCTGGTGCGTGGGGGGGGGTGGTGCG<br>Assembly 2: 266170-266199: GGTCGGCTGGTGCGT-GGGGGGGGTGGTGCG<br>*	Intergenic region<br>Intergenic region	The context is the same as in the case of the intergenic region in the strain TL110 (mismatch no. 3), the difference introduced into Assembly 2 by polishing.	1

	2	Assembly 1: 289783-289813: GTGGGTCGTCGGGCTGGGGGGCGTCGGGTCA<br>Assembly 2: 289782-289811: GTGGGTCGTCGGGCT-GGGGGCGTCGGGTCA<br>*	Anionic cell wall polymer biosynthesis enzyme TagV/TagU, LytR-Cps2A-Psr (LCP) family<br>Cell envelope-related transcriptional attenuator	The difference introduces frameshift resulting in a shorter ORF in the Assembly 2 with a product truncated at N-terminus.	1
	3	Assembly 1: 414312-414342: GGTGTTGGGGTGTGCGGGGGGGGGGCTGGGC<br>Assembly 2: 414310-414339: GGTGTTGGGGTGTGC-GGGGGGGGGCTGGGC<br>*	Hypothetical protein<br>Hypothetical protein	It is the same ORF as in the strain TL110 (mismatch no. 2), but different region. This region is identical in TL110 and TL29, but differs in other *P. freudenreichii* genomes, including TL19.	Inconclusive
	4	Assembly 1: 415024-415054: CTGTCCCTCCGGAATCCCCCCCCGGAATCCC<br>Assembly 2: 415021-415050: CTGTCCCTCCGGAAT-CCCCCCCGGAATCCC<br>*	Hypothetical protein<br>Hypothetical protein	It is the same ORF as in the strain TL110 (mismatch no. 2), but different region. The Assembly 1 version is conserved among other *P. freudenreichii* genomes, including the original HGAP assemblies of strains TL110 and TL19. The difference was introduced into Assembly 2 by polishing.	1
	5	Assembly 1: 1077164-1077194: CGACGTGCAGCATGGCCCCGGGGTGATCGTG<br>Assembly 2: 1077158-1077187: CGACGTGCAGCATGG-CCCGGGGTGATCGTG<br>*	Intergenic region<br>Intergenic region	The ONT variant is widespread in *P. freudenreichii* genomes and the difference was introduced to Assembly 2 by polishing.	1
	6	Assembly 1: 1265844-1265874: CGGGTGGGGGGTGTTGGGGGGATTTCCCGAA<br>Assembly 2: 1265837-1265866: CGGGTGGGGGGTGTT-GGGGGATTTCCCGAA<br>*	Intergenic region<br>Intergenic region	Region downstream of Glucan phosphorylase (as opposed to upstream as in mismatch no. 3 in the strain TL19). The ONT variant is widespread in *P. freudenreichii* genomes and the difference was introduced to the Assembly 2 by polishing.	1
	7	Assembly 1: 1600470-1600500: CCACCTTCGACGGCTCCCCCCCAAAAGGTTG<br>Assembly 2: 1600462-1600491: CCACCTTCGACGGCT-CCCCCCAAAAGGTTG<br>*	16S ribosomal RNA<br>16S ribosomal RNA	The same context as in the strain TL110 (mismatch no. 1).	1
	8	Assembly 1: 1828032-1828062: AGGGCCCCGCCCCGACCCCCCGACGGCGTCG<br>Assembly 2: 1828023-1828052: AGGGCCCCGCCCCGA-CCCCCGACGGCGTCG<br>*	DUF2510 domain-containing protein<br>DUF2510 domain-containing protein	The difference splits the PB gene into two truncated versions of the gene. Introduced into Assembly 2 by polishing.	1

Table S4. Accession numbers for strains TL110, TL19, and TL29 across different sequencing strategies.

Isolate	Library preparation	Flowcell	SRA accession no.
TL110	ONT Native (dark)	R10.4.1	SRR30427041
TL110	ONT Native (light)	R10.4.1	SRR27779106
TL110	ONT Rapid	R9.4.1	SRR27779095
TL110	ONT Rapid	R10.4.1	SRR30427045
TL110	ONT BARSEQ	R10.4.1	SRR30427044
TL110	Illumina	-	SRR27779094
TL110	PacBio	-	SRS10712865
TL110	ONT Native (dark)	R10.4.1	SRR30427040
TL19	ONT Native (dark)	R10.4.1	SRR30427037
TL19	ONT Native (light)	R10.4.1	SRR27779092
TL19	ONT Rapid	R9.4.1	SRR27779105
TL19	ONT Rapid	R10.4.1	SRR30427039
TL19	ONT BARSEQ	R10.4.1	SRR30427038
TL19	Illumina	-	SRR27779104
TL19	PacBio	-	SRS10717466
TL19	ONT Native (dark)	R10.4.1	SRR30427036
TL29	ONT Native (dark)	R10.4.1	SRR30427043
TL29	ONT Native (light)	R10.4.1	SRR27779102
TL29	ONT Rapid	R9.4.1	SRR27779099
TL29	ONT Rapid	R10.4.1	SRR30427035
TL29	ONT BARSEQ	R10.4.1	SRR30427034
TL29	Illumina	-	SRR27779098
TL29	PacBio	-	SRS10725002
TL29	ONT Native (dark)	R10.4.1	SRR30427042
